# Supplementary material for: Optogenetic Tuning Reveals Rho Amplification-Dependent Dynamics of a Cell Contraction Signal Network
Source: Cell Rep. 2020 Dec 1;33(9):108467. doi: 10.1016/j.celrep.2020.108467 (PMC7710677; doi:10.1016/j.celrep.2020.108467)
Supplement: Document S1. Figures S1–S4 and Tables S1–S3 [file mmc1.pdf]

Cell Reports, Volume *33*

Supplemental Information

Optogenetic Tuning Reveals Rho Amplification-Dependent Dynamics of a Cell Contraction Signal Network

Dominic Kamps, Johannes Koch, Victor O. Juma, Eduard Campillo-Funollet, Melanie Graessl, Soumya Banerjee, Tomáš Mazel, Xi Chen, Yao-Wen Wu, Stephanie Portet, Anotida Madzvamuse, Perihan Nalbant, and Leif Dehmelt

Supplemental Information

Supplemental Figures:

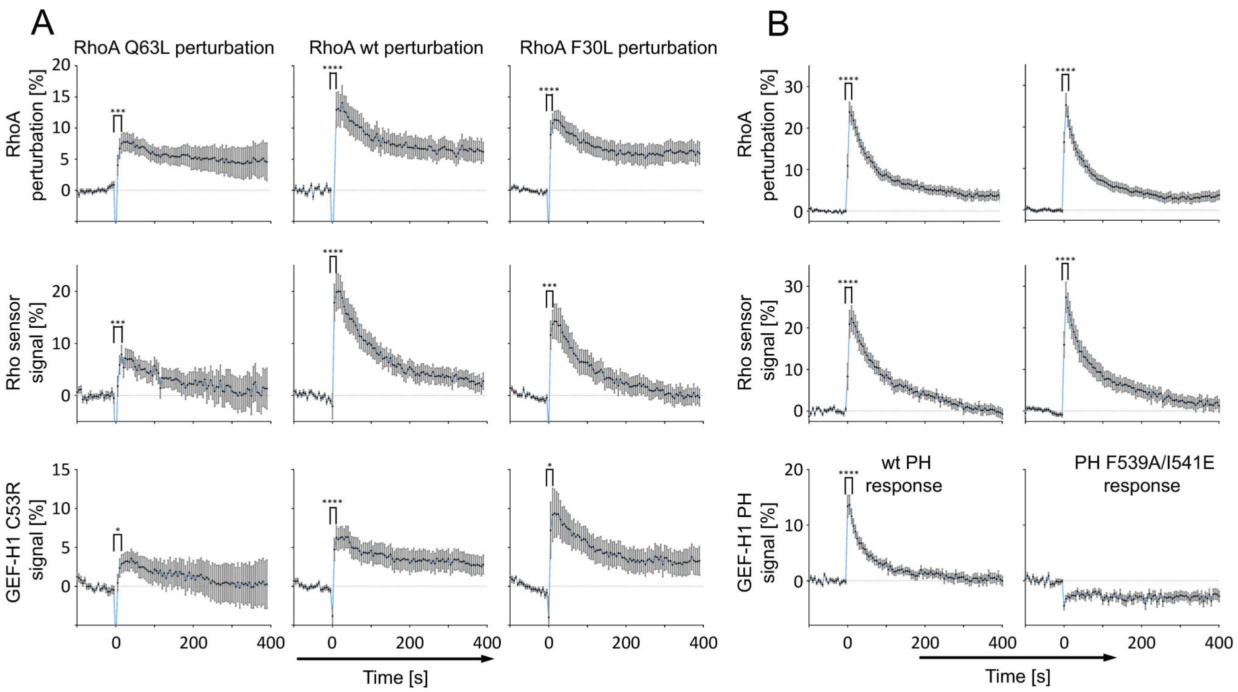

Supplemental Figure 1 (related to Figure 1): Direct investigation of Rho-dependent GEF-H1 plasma membrane recruitment. A: Quantification of the RhoA plasma membrane recruitment and Rho activity sensor/GEF-H1 co-recruitment. **B:** Quantification of GEF-H1 PH domain co-recruitment with active RhoA Q63L. F539A/I541E: PH domain mutant that is deficient in its interaction with active Rho. (**A-B**: % increase above average intensity before photoactivation at t=0s with standard error of the mean (SEM); $n \geq 14$ (**A**) or $n \geq 21$ (**B**) cells from three experiments; ****, $P \leq 0.0001$; ***, $P \leq 0.001$; **, $P \leq 0.01$; *, $P \leq 0.05$; paired t test before and 10 s after photoactivation);

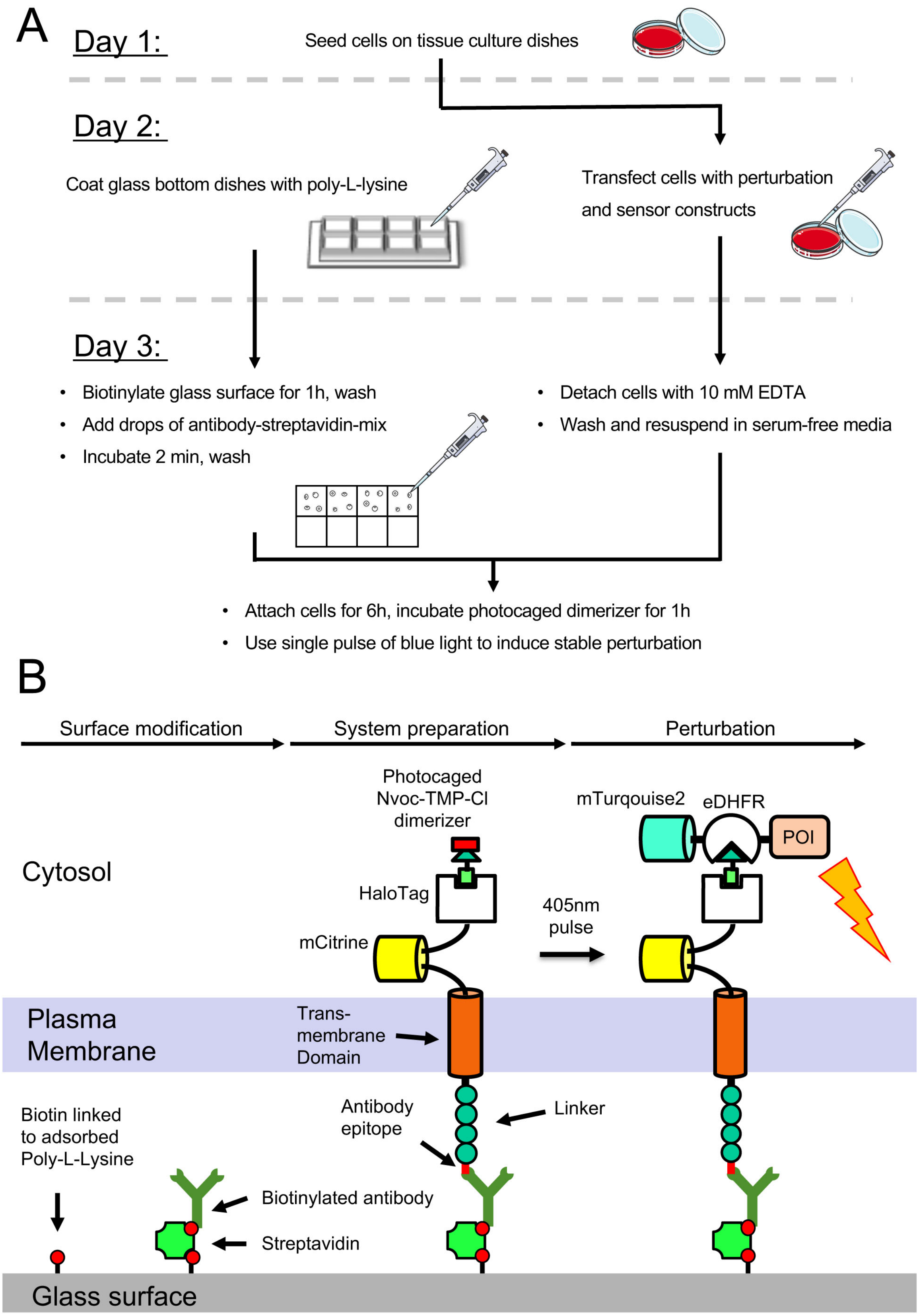

Supplemental Figure 2 (related to Figure 2): Schematic summary of improved "Molecular Activity Painting" technique to acutely introduce stable perturbations at the plasma membrane of living cells. A: Experimental workflow and time considerations. **B**: Schematics for step-wise construction of perturbation system. This figure was created using Servier Medical Art templates, which are licensed under a Creative Commons Attribution 3.0 Unported License; https://smart.servier.com

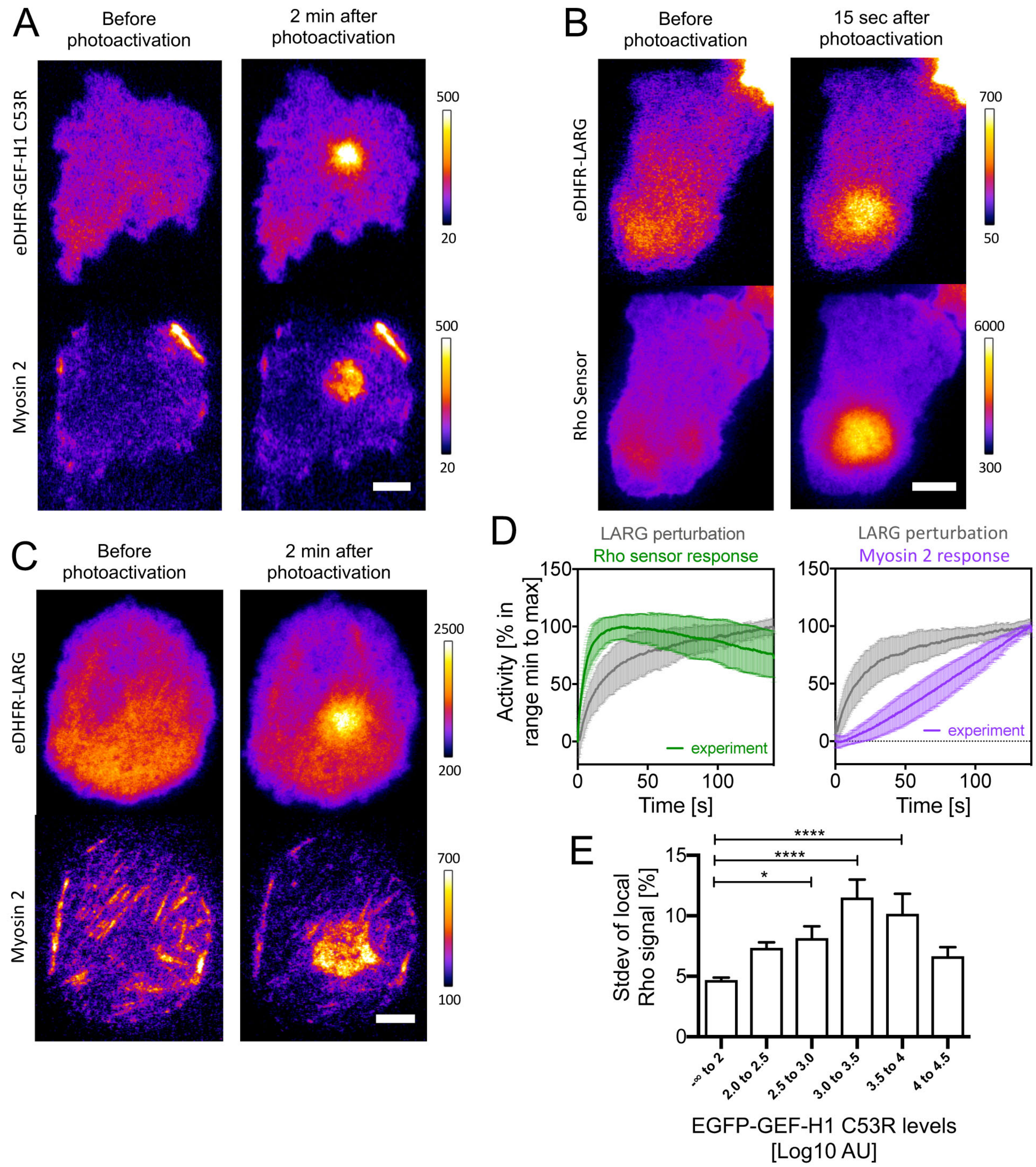

Supplemental Figure 3 (related to Figure 2): Rho activity and myosin cell cortex recruitment response to Lbc-type GEF perturbation. A: TIRF images of immobilized GEF-H1 C53R perturbation and Myosin response (see also Video 2). **B-C:** TIRF images of immobilized LARG perturbation and Rho activity sensor **(B)** or Myosin **(C)** response (see also Video 2). **D:** Kinetics of Rho activity sensor and Myosin cell cortex recruitment response to acute chemo-optogenetic LARG perturbations ($n \geq 23$ cells from three experiments, mean with SEM). **E**: Dependence of Rho activity dynamics on the effective cytosolic concentration of GEF-H1 C53R. Activity dynamics were measured by determining the standard deviation of the local Rho activity sensor signal (n=124 cells from three experiments; mean and SEM; One way ANOVA, Dunnett's post test, *: P<0.05, ****: P<0.0001). % indicates percentage of mean intensity. Scale bars: 10µm.

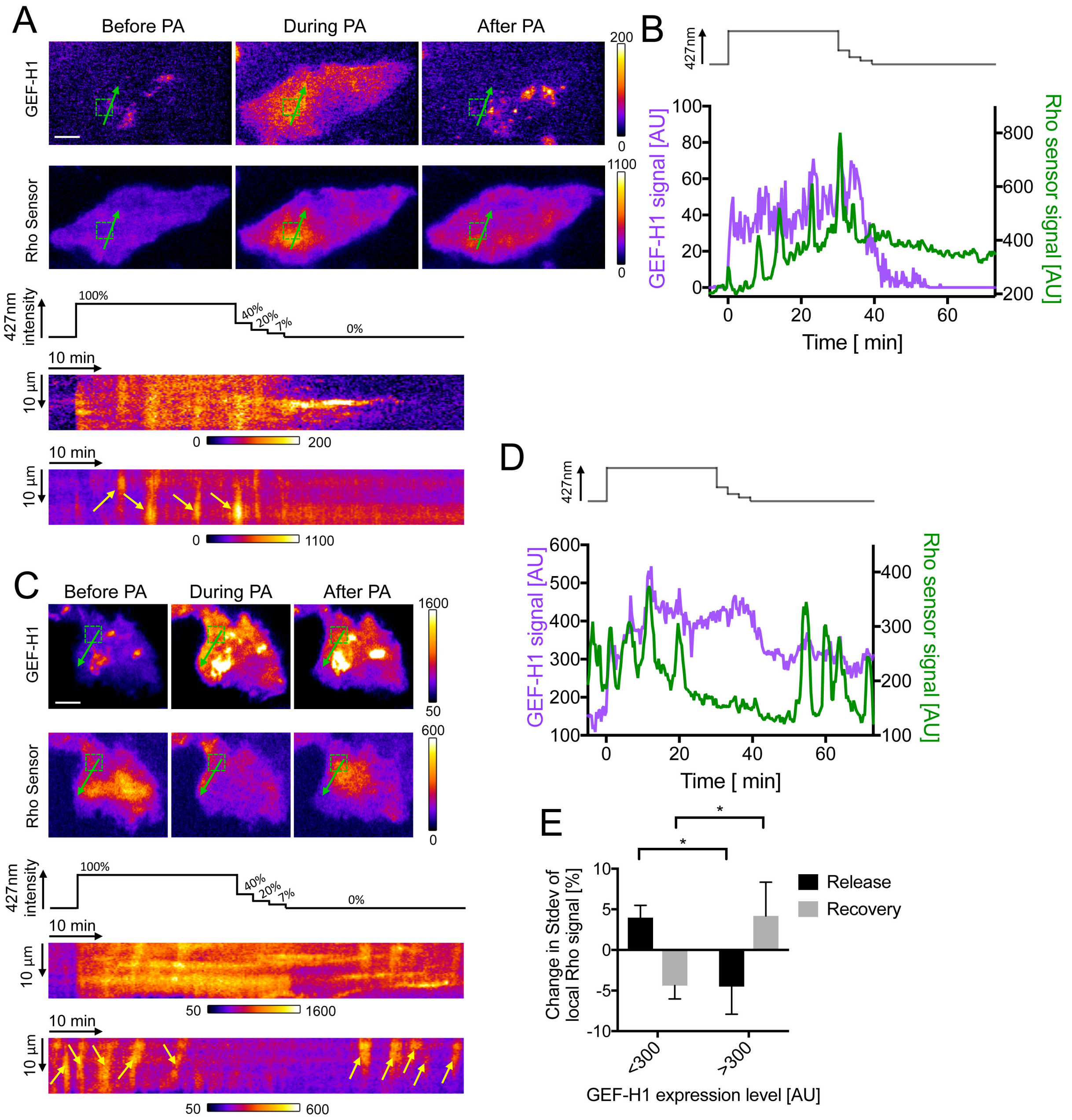

Supplemental Figure 4 (related to Figure 3): GEF-H1 concentration-dependent switching of Rho activity dynamics by reversible optogenetic tuning. A-D: Analysis of two representative cells that demonstrate either reversible activation **(A,B)** or reversible saturation **(C,D)** of Rho activity dynamics during subsequent increase and decrease of the effective cytosolic concentration of GEF-H1 via photoactivation (PA) of the LOVTRAP system. **A,C:** Top: TIRF images of mCherry-Zdk1-GEF-H1(C53R) and the Rho sensor (see also Video 5). Bottom: Kymographs corresponding to green arrows in top panel. Yellow arrows point to Rho activity pulses or Rho activity waves. **B,D:** Cytosolic mCherry-Zdk1-GEF-H1(C53R) levels obtained as the minimum signal in green boxed regions in **A** or **C**, and measurements of the Rho activity sensor signal over the time course of the experiment. **E:** Local standard deviation of Rho activity signals at low (<300) or high (>300) levels of mCherry-Zdk1-GEF-H1(C53R) from all analyzed cells (n = 16 cells from three experiments, mean and SEM; unpaired t-test, *: P<0.05). Columns represent the change of the average local standard deviation of Rho activity signals induced by the release of GEF-H1 from mitochondria (comparing the time ranges -5 to 0 min before PA with 25 – 30 min during PA) or induced by the recovery of GEF-H1 to mitochondria (comparing the time ranges 25 – 30 min during PA with 25 – 30 min after PA). % indicates percentage of mean intensity. PA: photoactivation. Scale bars: 10µm.

Supplemental tables

Experimental measurement	Measured values
GEF-H1 perturbation rate constant (mono-exponential fit to grey curve in Figure 2D)	0.0789 +/- 0.006 s^{-1} (with Rho sensor; left panel)<br>0.0397 +/- 0009 s^{-1} (with Myosin; right panel)
Rho and Myosin response kinetics	Simulations were fitted to all time points shown in Figure 2D
Period of Rho activity pulses	248 +/- 95 s (n=10 cells; in the presence of active GEF-H1 C53R, this work)
Time shift Myosin after Rho	39.5 +/- 14.7 s (n=37 cells; Graessl et al.)
Time shift Rho after GEF-H1	2.5 +/- 5.6 s (n=68 cells; Graessl et al.)

Supplemental Table 1: Experimental data used to parameterize the ODE system (related to Figure 2). The ODE system is given in Eq.(1-3).

Parameter	Lower bound for fitting	Upper bound for fitting
Concentrations		
in 10^6 molecules/cell:		
G_T (oscillatory regime)	0.142	20
G_T (perturbation/Rho response)	0.0142	1
G_T (perturbation/Myosin response)	0.0142	1
R_T (all regimes; fixed value based on[12])	0.443	0.443
M_T (all regimes; fixed value based on[12])	1.24	1.24
Rate constants:		
k_1/K_{m1} in $s^{-1}(10^6$ molecules/cell$)^{-2}$	0.316	31.6
k_2/K_{m2} in $s^{-1}(10^6$ molecules/cell$)^{-1}$	0.15	150
k_3 in $s^{-1}(10^6$ molecules/cell$)^{-2}$	0.15	15
k_4 in $s^{-1}(10^6$ molecules/cell$)^{-2}$	0.015	15
k_5/K_{m5} in $s^{-1}(10^6$ molecules/cell$)^{-2}$	0.005	5.00
k_6/K_{m6} in $s^{-1}(10^6$ molecules/cell$)^{-1}$	0.000844	2.67
Michaelis constants		
in 10^6 molecules/cell:		
K_{m1}	0.0475	47.5
K_{m2}	0.01	10
K_{m5}	0.003	3.00
K_{m6}	0.0563	5.63

Supplemental Table 2: Parameters of the ODE system and their prior distributions for fitting to experimental data (related to Figure 2). The ODE system is given by Eq.(1-3). The distributions are defined by the given ranges with equal probability.

Physical parameter	Estimated value (mode of distribution)	95% lower bound	95% upper bound
Total concentrations in 10^6 molecules/cell:			
GEF-H1 (G_T); oscillatory regime	0.179	0.178	4.64
GEF-H1 (G_T); perturbation regime; Rho response	0.0858	0.0491	0.175
GEF-H1 (G_T); perturbation regime; Myosin response	0.233	0.0203	0.234
Rho (R_T); all regimes; fixed value based on[12]	0.443		
Myosin (M_T); all regimes; fixed value based on[12]	1.24		
Rate constants:			
k_1/K_{m1} in $s^{-1}(10^6$ molecules/cell$)^{-1}$	3.88	1.09	5.37
k_2/K_{m2} in s^{-1}	2.04	0.880	3.83
k_3 in $s^{-1}(10^6$ molecules/cell$)^{-1}$	1.19	0.723	3.34
k_4 in $s^{-1}(10^6$ molecules/cell$)^{-1}$	3.98	3.97	14.9
k_5/K_{m5} in $s^{-1}(10^6$ molecules/cell$)^{-1}$	0.417	0.074	0.718
k_6/K_{m6} in s^{-1}	0.00509	0.000904	0.00510
Michaelis constants in 10^6 molecules/cell:			
K_{m1}	2.42	0.0886	2.43
K_{m2}	0.0745	0.0741	0.564
K_{m5}	0.014	0.00733	0.0658
K_{m6}	0.786	0.258	1.83

Supplemental Table 3: Parameters of the ODE system and their estimations obtained via fitting to experimental data (related to Figure 2). The ODE system is given by Eq.(1-3). The values correspond to the mode of the posterior distribution. The lower and upper bounds correspond to the highest posterior density region, containing 95% of the posterior probability mass.
