# Supplementary material for: Optogenetic Tuning Reveals Rho Amplification-Dependent Dynamics of a Cell Contraction Signal Network
Source: Cell Rep. 2020 Dec 1;33(9):108467. doi: 10.1016/j.celrep.2020.108467 (PMC7710677; doi:10.1016/j.celrep.2020.108467)
Supplement: Document S2. Article plus Supplemental Information [file mmc9.pdf]

Cell Reports

Article

Optogenetic Tuning Reveals Rho Amplification-Dependent Dynamics of a Cell Contraction Signal Network

Graphical Abstract

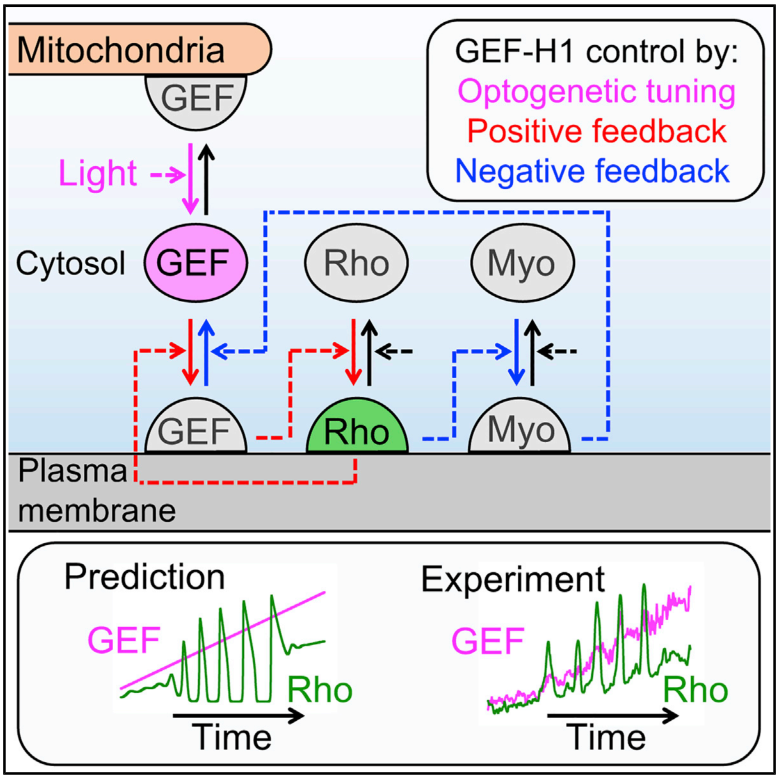

Highlights

- Cell contraction dynamics are investigated via acute chemo-optogenetic perturbations
- A theoretical model predicts GEF-H1-dependent switches in pulsatile Rho dynamics
- Experimental manipulations of cytosolic GEF-H1 levels confirm model predictions
- Local pulsatile dynamics are generated by coupling feedback regulation and diffusion

Authors

Dominic Kamps, Johannes Koch, Victor O. Juma, ..., Anotida Madzvamuse, Perihan Nalbant, Leif Dehmelt

Correspondence

leif.dehmelt@mpi-dortmund.mpg.de

In Brief

Kamps et al. derive a reaction-diffusion system that generates subcellular activity pulses of the cell contraction regulator Rho. System parameters are obtained by combining perturbation-response analyses with theoretical investigations. Multiple switches in activity dynamics, which are dependent on the positive feedback mediator GEF-H1, are predicted by theory and confirmed experimentally.

Kamps et al., 2020, Cell Reports *33*, 108467
December 1, 2020 © 2020 The Authors.
https://doi.org/10.1016/j.celrep.2020.108467

CellPress

Cell Reports

CellPress
OPEN ACCESS

Article

Optogenetic Tuning Reveals Rho Amplification-Dependent Dynamics of a Cell Contraction Signal Network

Dominic Kamps,[1,2] Johannes Koch,[3] Victor O. Juma,[4] Eduard Campillo-Funollet,[4] Melanie Graessl,[3] Soumya Banerjee,[1,2] Tomáš Mazel,[1,2] Xi Chen,[5,6] Yao-Wen Wu,[5,7] Stephanie Portet,[8] Anotida Madzvamuse,[4,9,10] Perihan Nalbant,[3] and Leif Dehmelt[1,2,11,*]

[1]Department of Systemic Cell Biology, Max Planck Institute of Molecular Physiology, 44227 Dortmund, Germany
[2]Faculty of Chemistry and Chemical Biology, TU Dortmund University, 44227 Dortmund, Germany
[3]Department of Molecular Cell Biology, Center for Medical Biotechnology, University of Duisburg-Essen, 45141 Essen, Germany
[4]Department of Mathematics, University of Sussex, Pevensey III, Brighton BN1 9QH, UK
[5]Chemical Genomics Centre of the Max-Planck Society, 44227 Dortmund, Germany
[6]The HIT Center for Life Sciences, Harbin Institute of Technology, Harbin, P.R. China
[7]Department of Chemistry, Umeå Centre for Microbial Research, Umeå University, 901 87 Umeå, Sweden
[8]Department of Mathematics, University of Manitoba, Winnipeg, MB R3T 2N2, Canada
[9]Department of Mathematics, University of Johannesburg, South Africa
[10]Universita degli Studi di Bari Aldo Moro, Bari, Italy
[11]Lead Contact
*Correspondence: leif.dehmelt@mpi-dortmund.mpg.de
https://doi.org/10.1016/j.celrep.2020.108467

SUMMARY

Local cell contraction pulses play important roles in tissue and cell morphogenesis. Here, we improve a chemo-optogenetic approach and apply it to investigate the signal network that generates these pulses. We use these measurements to derive and parameterize a system of ordinary differential equations describing temporal signal network dynamics. Bifurcation analysis and numerical simulations predict a strong dependence of oscillatory system dynamics on the concentration of GEF-H1, an Lbc-type RhoGEF, which mediates the positive feedback amplification of Rho activity. This prediction is confirmed experimentally via optogenetic tuning of the effective GEF-H1 concentration in individual living cells. Numerical simulations show that pulse amplitude is most sensitive to external inputs into the myosin component at low GEF-H1 concentrations and that the spatial pulse width is dependent on GEF-H1 diffusion. Our study offers a theoretical framework to explain the emergence of local cell contraction pulses and their modulation by biochemical and mechanical signals.

INTRODUCTION

Cells can sense various physical and chemical signals from their environment to steer changes in their dynamic behavior (Kim et al., 2018; Saha et al., 2018). This sensing process is particularly important during embryogenesis, in which the differentiation and migration of cells is controlled both by chemical morphogens and the mechanical properties of the extracellular matrix (Kim et al., 2018; Saha et al., 2018). The sensing of chemical signals, such as growth factor concentration, is primarily a passive process, in which a diffusible molecule engages a corresponding cellular receptor. In contrast, probing of mechanical signals requires an active process that generates force to deform physical structures that interact with cells (Kim et al., 2018; Nalbant and Dehmelt, 2018; Saha et al., 2018). For example, the differentiation of stem cells is steered by the elasticity of the cell environment, which differs significantly between tissues such as brain or bone (Engler et al., 2006). This mechanosensing process depends on myosin motors (Engler et al., 2006) that produce contractile forces.

Interestingly, myosin-generated contractile forces are often pulsatile (Baird et al., 2017; Bement et al., 2015; Coravos et al., 2017; Graessl et al., 2017; Maître et al., 2015; Munjal et al., 2015; Nalbant and Dehmelt, 2018; Nishikawa et al., 2017; Wu, 2017). Such pulsed contractions enable efficient remodeling of tissues in various contexts during development (Gorfinkiel and Blanchard, 2011; Martin et al., 2009). For example, during apical constriction, pulsatile contractions enable a ratcheting mechanism to drive large-scale cell rearrangements (Martin et al., 2009). By generating sequential, shorter pulses, cells can repeatedly and locally probe the elasticity of their environment (Nalbant and Dehmelt, 2018; Plotnikov et al., 2012), and this process was proposed to play a role in probing local differences in substrate elasticity during durotaxis (Plotnikov et al., 2012). Cells are more sensitive to external cyclic stretch compared to constant counterforces (Cui et al., 2015), and thus a pulsatile intracellular force

This is an open access article under the CC BY license (http://creativecommons.org/licenses/by/4.0/).

generating mechanism may also be more efficient in transducing external mechanical cues (Nalbant and Dehmelt, 2018).

We recently proposed a mechanosensitive process in adherent, mammalian cells that involves such local pulses of myosin-dependent cell contraction that are controlled by the small guanosine triphosphatase (GTPase) Rho (Graessl et al., 2017). In this system, the frequency of local contraction pulses was modulated by the elasticity of the extracellular matrix (Graessl et al., 2017). This shows that extracellular mechanical signals are transduced into a change in system dynamics; however, a theoretical concept that describes how these dynamics are generated and how they are modulated by biochemical and mechanical inputs was missing.

Here, we used acute protein activity perturbation to directly investigate causal relationships between key components that control local cell contraction pulses. Based on our experimental investigations, we derived a quantitative model for signal network dynamics. A theoretical analysis predicted switches between distinct dynamic states that are dependent on the concentration of the Rho-activating Lbc-type GEF GEF-H1 (ARHGEF2). We confirmed these predictions by optogenetic tuning of the cytosolic concentration of GEF-H1 in individual living cells. By combining experimental investigations and numerical simulations of the system dynamics, we developed a theoretical framework for spatiotemporal cell contraction pattern formation and its modulation by biochemical or mechanical inputs.

RESULTS

Direct Experimental Investigation of Rho Amplification

Depolymerization of microtubules by nocodazole treatment increases the cytosolic concentration of the Rho-activating regulator GEF-H1 (Chang et al., 2008; Krendel et al., 2002). Interestingly, this increased concentration does not simply elevate basal Rho activity, but instead dramatically stimulates Rho activity dynamics (Figures 1A and 1B) (Graessl et al., 2017). To investigate this dynamic system, we analyzed the interplay between GEF-H1 and Rho activity.

To induce acute RhoA activity perturbations, we applied a variant of a chemo-optogenetic technique that we recently developed (Chen et al., 2017) (Figure 1C). In this variant, we used a pulse of light to uncage a chemical dimerizer that is covalently linked to a plasma membrane anchor. This uncaging event enables plasma membrane targeting of E. coli dihydrofolate reductase (eDHFR) fusion proteins of Rho. We combined this acute perturbation method with total internal reflection fluorescence (TIRF) microscopy-based readouts of a Rho activity probe to experimentally measure how plasma membrane targeting of eDHFR fusion proteins affect Rho activity (Figure 1C). To measure Rho activity, we used a low-expressing, fluorescently tagged GTPase binding domain (GBD) that selectively binds the active form of Rho (Graessl et al., 2017). Acute, local targeting of constitutively active Rho (eDHFR-RhoA Q63L) was expected to induce a local increase in Rho activity. We observed a robust increase in the Rho activity sensor signal at the perturbation site (Figures 1D and 1E; Video S1). Targeting of wild-type Rho or the fast-cycling mutant F30L also increased Rho activity signals, suggesting that at least a fraction of these constructs was in the active state (Figure S1A). The Rho sensor response to wild-type or fast-cycling Rho appeared to be even stronger compared to constitutively active Rho. This may be due to a higher basal Rho activity sensor signal that is caused by constitutively active Rho, resulting in a lower dynamic range in the response signal. Interestingly, GEF-H1 was co-recruited together with these Rho constructs and the Rho activity sensor (Figures 1D, 1E, and S1A; Video S1). This clearly demonstrates that a local increase in Rho activity causes increased plasma membrane recruitment of GEF-H1.

It was previously shown that the Pleckstrin homology (PH) domain of GEF-H1 and related Lbc-type GEFs can interact with active Rho (Medina et al., 2013). We find that this domain is sufficient for plasma membrane recruitment to increased Rho activity at local perturbation sites (Figure 1F). In contrast, the dominant-negative, inactive Rho mutant T19N cannot recruit the GEF-H1 PH domain to the plasma membrane. This shows that the interaction with the GEF-H1 PH domain is dependent on the Rho activity state (Figure 1F). Introducing two point mutations within the PH domain of GEF-H1 that are localized to the Rho interaction surface (Medina et al., 2013) interferes with its plasma membrane recruitment to active Rho (Figure S1B). This shows that an intact PH domain is both necessary and sufficient for Rho activity-dependent GEF-H1 plasma membrane recruitment. Together with the well-documented ability of the Dbl homology (DH) domain to activate Rho via nucleotide exchange (Rossman et al., 2005), this recruitment activity closes a positive feedback loop that can amplify Rho activity in cells.

Derivation and Parameterization of a Quantitative Temporal Model for Cell Contraction Signal Network Dynamics

To gain a more quantitative understanding of Rho activity dynamics, we formulated a scheme that describes the most relevant biochemical reactions (Figure 2A; see Method Details for justification of their specific implementation). The most important features of this system are the positive feedback loop between Rho and the Lbc family member GEF-H1 (Figure 1) and a negative feedback loop that acts via inhibition of GEF-H1 via myosin (non-muscle myosin-IIa, MYH9; Graessl et al., 2017; Lee et al., 2010). The active forms of Rho and myosin are known to be localized preferably near the plasma membrane, either via direct membrane association or the submembraneous actin cortex, whereas the inactive forms are predominantly cytosolic (Citi and Kendrick-Jones, 1987; Garcia-Mata et al., 2011). The motor domain of active myosin was shown to bind the DH domain of Dbl family GEFs, which includes GEF-H1, thereby inhibiting its nucleotide exchange activity (Lee et al., 2010). The inactivation of GEF-H1 would lead to less activation of Rho, which targets GEF-H1 to the plasma membrane. As these processes happen on a faster timescale than the overall system dynamics, the association and dissociation of GEF-H1 are expected to closely follow Rho activity. Thus, the activity state of the three major signal network components, GEF-H1, Rho, and myosin, is expected to drive dynamic plasma membrane association/dissociation kinetics (Figure 2A).

We then used the biochemical reaction scheme to derive a system of three ordinary differential equations (ODEs) that

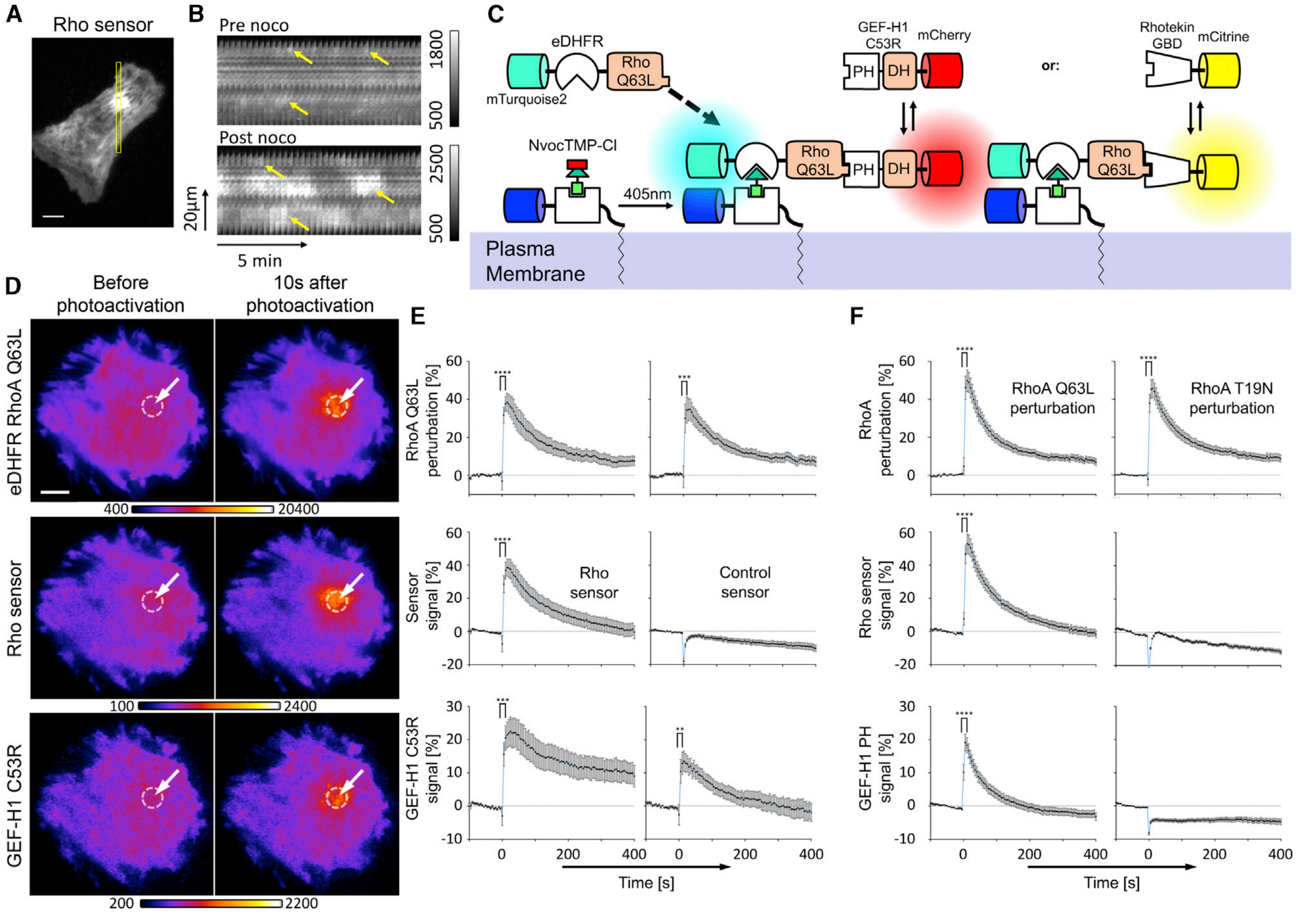

Figure 1. Direct Investigation of Rho-Dependent GEF-H1 Plasma Membrane Recruitment

(A) TIRF image of a U2OS cell that expresses the Rho activity sensor mCherry-Rhotekin-GBD. Rho activity dynamics were stimulated by releasing GEF-H1 (ARHGEF2) from microtubules into the cytosol via nocodazole.

(B) Kymographs corresponding to the yellow box in (A), which represent irregular, Rho activity dynamics before and after nocodazole application.

(C) Schematic representation of acute chemo-optogenetic plasma membrane recruitment of active Rho. NvocTMP-Cl: photocaged chemical dimerizer; HaloTag/eDHFR: dimerization domains for chemo-optogenetic perturbation.

(D) Representative TIRF images of chemo-optogenetic mTurquoise2-eDHFR-Rho Q63L plasma membrane recruitment and co-recruitment of Rho activity sensor (mCitrine-Rhotekin-GBD) and cytosolic, microtubule-binding deficient GEF-H1(C53R) mutant fused to mCherry (see also Video S1).

(E and F) Quantification of co-recruitment of RhoA constructs, Rho activity or control sensors, and GEF-H1(C53R) or GEF-H1 PH domain (percentage increase above average intensity before photoactivation at t = 0 s with standard error of the mean (SEM); n $\geq$ 10 (E) or n $\geq$ 15 (F) cells from 3 experiments; **p $\leq$ 0.01; ***p $\leq$ 0.001; ****p $\leq$ 0.0001; paired t test before and 10 s after photoactivation.

Scale bars: 10 µm.

See also Figure S1.

describes the temporal dynamics of the three system components: active GEF, active Rho, and active myosin (see Equations 1, 2, and 3 in Method Details). To characterize the contribution of these biochemical reactions to the system dynamics, we studied a model that is homogeneous in space. The ODE model includes 13 parameters, which are the component total concentrations, reaction rates, and Michaelis constants. The total concentrations of Rho and myosin were derived from mass spectrometry-based analyses performed in U2OS cells (Beck et al., 2011). To estimate the remaining 11 free parameters, we performed detailed quantitative measurements of the system dynamics.

To directly measure the kinetics of the central reactions in this signal network, we developed an improved generic variant of an acute, chemo-optogenetic signal network perturbation technique that we recently developed. The original method, which we called molecular activity painting (Chen et al., 2017), enabled switch-like perturbations of signal networks at the plasma membrane within seconds. The improved variant that we introduce in this study (Figures 2B and S2; Method Details) does not require specialized DNA-directed immobilization or surface modification procedures. Instead, glass surfaces are functionalized by a very simple protocol that is based on surface adsorption.

We applied this improved method to acutely target an active, microtubule-binding deficient mutant of GEF-H1 (C53R) to the plasma membrane. We then measured how quickly this perturbation induced an increase in the Rho activity sensor (Figures

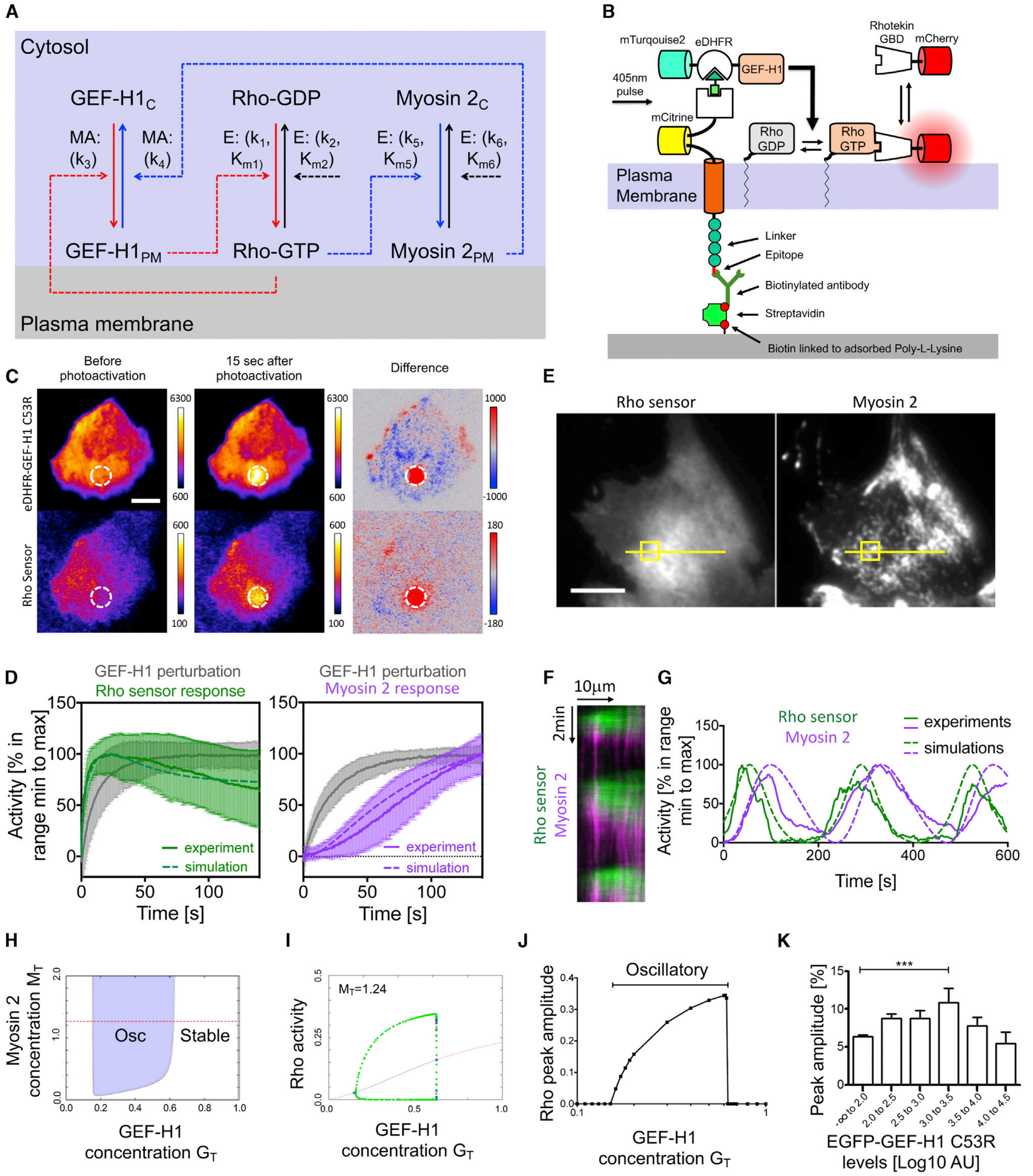

Figure 2. Parameterization and Analysis of a Temporal Model for Cell Contraction Signal Network Dynamics

(A) A biochemical reaction scheme for positive and negative feedback regulation of Rho activity. GEF-H1 mediates positive feedback (highlighted in red) and myosin (non-muscle myosin-IIa, MYH9) mediates negative feedback (highlighted in blue). E, enzymatic reaction, MA, mass action, $_{C}$, cytosolic, $_{PM}$, plasma membrane-associated (a detailed description of the model and a justification of the specific implementation is given in Method Details).

(B) A schematic representation of improved molecular activity painting, a simplified, generic method for immobilized chemo-optogenetic plasma membrane recruitment and its application to introduce acute and stable GEF-H1 perturbations (see Method Details).

(legend continued on next page)

2C and 2D; Table S1; Video S2). These experiments revealed that Rho activation occurs very rapidly within a few seconds after the chemo-optogenetic perturbation. In contrast, recruitment of the negative feedback mediator myosin occurred much slower over the time course of minutes (Figures 2D and S3A; Video S2).

It should be noted that the Rho activity response quickly reaches a maximum and subsequently decays to a lower level with slower kinetics. These two phases were expected due to the proposed rapid positive feedback and delayed negative feedback regulation. GEFs from the related Lbc family are expected to share these properties, as they can bind to active Rho (Medina et al., 2013) and also belong to the Dbl family that is inhibited by myosin (Lee et al., 2010). We found earlier that increased expression of the Lbc-GEF LARG (ARHGEF12) stimulates Rho activity pulses similarly to GEF-H1 (Graessl et al., 2017). Here, we find that acute targeting of LARG to the plasma membrane induced a biphasic response similar to that of GEF-H1, which further supports this idea (Figures S3B–S3D; Video S2).

In addition to measuring the kinetics of the signal network response to acute perturbations, we measured spontaneous activity dynamics that are stimulated by increasing cytosolic GEF-H1 levels either by overexpression of the microtubule-binding deficient C53R mutant (Table S1) or by applying nocodazole (Graessl et al., 2017) (Figures 2E–2G; Video S3). The period of Rho activity was determined by fast Fourier transformation (FFT) analysis, and the width of pulses and time lags between Rho and myosin were measured via time series and cross-correlation analysis (Graessl et al., 2017) (Table S1).

These experimental measurements were then used to fit the 11 free parameters of the ODE system. To maximally constrain the free parameters, we simultaneously fitted ODE simulations to experiments in 2 regimes: (1) Rho and myosin activation after acute GEF-H1 perturbation at a low total concentration of GEF-H1 (perturbation regime; Figure 2D) and (2) spontaneous oscillatory system dynamics at increased concentrations of total GEF-H1 (oscillatory regime; Figure 2G). As we neglect spatial information, the ODE model represents local dynamics over time. Therefore, we fitted ODE model simulations to measurements of activity dynamics within small regions of the plasma membrane (circles in Figures 2C and 2E). Except for the total concentration of GEF-H1, all of the parameters were kept identical in these two regimes. To search this parameter space, we used a Bayesian approach and a parallelized Markov chain Monte Carlo method (MCMC) (Campillo-Funollet et al., 2019) starting from 15 distinct sets of parameters, which were randomly sampled from uniform prior distributions (see Table S2 for parameter ranges and Method Details). Table S3 shows the parameter values with the highest probability (i.e., the mode of the posterior distributions, and the 95% credible intervals [CIs]).

Model Predictions: Dependence of System Dynamics on the Concentration of the Feedback Mediator GEF-H1

Numerical simulations performed using estimates of the parameter values exhibited major features of the observed system dynamics. First, simulations in the perturbation regime effectively represented experimentally measured Rho and myosin activation kinetics (Figure 2D). Second, simulations in the oscillatory regime generated system dynamics that closely mimicked experimentally measured width and time shifts of Rho, GEF, and myosin pulses (Figure 2G). This shows that the proposed biochemical reaction scheme (Figure 2A) and the corresponding system of ODEs are sufficient to generate the observed temporal system dynamics.

We next performed a detailed numerical analysis of the ODE system to predict how variations in the total concentration M_T of the negative feedback mediator myosin and G_T of the positive feedback mediator GEF-H1 affect Rho activity dynamics (Figures 2H and 2I). A two-component bifurcation analysis showed that oscillations only occur in concentration ranges, in which neither myosin nor GEF-H1 is zero. Thus, based on this proposed system, both positive and negative feedback are required for oscillatory system dynamics (Figure 2H). This is in agreement with our previous experimental observation that interfering with GEF-H1 or myosin inhibited Rho activity pulses (Graessl et al., 2017). This analysis also confirms our experimental observation that increased GEF-H1 concentrations stimulate oscillatory Rho activity dynamics (Figures 1A and 1B) (Graessl et al., 2017). At the lowest concentrations, the system is stable, and at increased GEF-H1 concentrations, the system can generate limit-cycle oscillations. In addition, the two-component (Figure 2H) and one-component (Figure 2I) bifurcation analyses also predicted an additional transition toward lower Rho activity dynamics at the

(C) TIRF images of immobilized GEF-H1(C53R) perturbation and Rho sensor response (see also Video S2).

(D) Kinetics of Rho activity sensor and myosin cell cortex recruitment response to acute chemo-optogenetic GEF-H1 perturbations and the corresponding numerical simulations of system dynamics using Equations 1, 2, and 3 ($n \geq 20$ cells from at least 3 experiments, means and SEMs).

(E) TIRF images of U2OS cells, in which cytosolic GEF-H1 levels were increased by nocodazole-induced microtubule depolymerization (see also Video S3).

(F) Kymograph corresponding to the yellow line shown in (E) to visualize regular pulses of Rho activity followed by myosin cell cortex recruitment.

(G) Quantification of experimentally measured Rho activity and myosin cell cortex recruitment pulse dynamics corresponding to the yellow box in (E), and corresponding numerical simulations of system dynamics.

(H) Two parameter bifurcation analysis of Equations 1, 2, and 3 to predict total concentration ranges of the positive and negative feedback mediators GEF-H1 and myosin, which can generate stable or oscillatory system dynamics.

(I) One parameter bifurcation analysis of Rho activity dynamics predicts limit cycle oscillations at intermediate GEF-H1 concentrations (subcritical Hopf bifurcations at $G_T = 0.1557$ and $G_T = 0.6215$).

(J) Simulations of Rho activity dynamics predict maximal Rho activity peak amplitude at intermediate GEF-H1 concentrations.

(K) Experimental confirmation of maximal Rho activity peak amplitude at intermediate GEF-H1(C53R) expression levels (percentage of signal above background; n = 124 cells from 3 experiments; means and SEMs; ***1-way ANOVA, Dunnett's post test, $p < 0.001$).

Scale bars: 10 μm.

See also Figures S2 and S3.

highest GEF-H1 concentrations, at which the system was again stable.

Further analysis using ODE simulations predicted that the peak Rho amplitude is minimal at low and high GEF-H1 concentrations and reaches a maximum at intermediate GEF-H1 concentrations (Figure 2J). Experimental manipulations of the GEF-H1 concentration confirmed this model prediction. The highest Rho activity pulses, as measured by the peak amplitude were found at intermediate expression levels of active GEF-H1 (Figure 2K). Using the standard deviation (SD) of the local signal (see Method Details) as an alternative measure for system dynamics, we corroborate this observation (Figure S3E), thereby further supporting our experimental confirmation of the model prediction.

Switching of Cell Contraction Signal Network Dynamics in Single Cells by Optogenetic Tuning of the Cytosolic GEF-H1 Concentration

Next, we performed simulations in which we gradually increased the total GEF-H1 concentration G_T to investigate how quickly the system dynamics respond to changes in this parameter. These simulations predict that Rho activity oscillations rapidly begin and end at GEF-H1 concentrations G_T that correspond to the subcritical Hopf bifurcations of the ODE system (Figures 2I and 3B).

To test this model prediction, we applied the LOVTRAP system (Wang et al., 2016), which enabled us to manipulate the effective, cytosolic concentration of GEF-H1 in individual living cells by releasing it from mitochondria (Figures 3A and 3C–3F). In this system, illumination with blue light led to the reversible release of the dark-state binding Zdk1 domain from light-oxygen-voltage-sensing (LOV2) domains that are anchored at mitochondria. In our experimental conditions, stepwise small increases in blue light intensity led to a continuous increase in the cytosolic levels of GEF-H1 (Figures 3C and 3D; Video S4) over time. This continuous increase was accompanied by a change in the system dynamics from very small activity pulses to high amplitude pulses, followed by recurring, propagating waves (Figures 3C and 3D; Video S4). Interestingly, at the highest cytosolic levels, wave propagation, and pulse amplitude diminished again, and the overall system dynamics were greatly reduced (Figures 3C–3E; Video S4). Direct comparison of theoretical (Figure 3B) and experimental (Figure 3D) tuning of the cytosolic GEF-H1 concentration shows very similar changes in Rho activity dynamics. Combining multiple cells and plotting a measure of Rho activity dynamics, the local SD of Rho sensor signal, against the effective cytosolic GEF-H1 signal (Figure 3F), confirmed the existence of three dynamic regimes: low activity amplitude in the stable regime at low GEF-H1 levels, high amplitude at intermediate GEF-H1 levels, and low amplitude at the highest GEF-H1 levels. The reduced Rho activity dynamics at the highest GEF-H1 levels suggests that this system is saturated under these conditions. To test whether switching system dynamics is reversible, we used the LOVTRAP system to sequentially increase and decrease the effective cytosolic GEF-H1 concentration (Figure S4; Video S5). We found that both the activation and saturation of system dynamics are reversible (Figure S4; Video S5). Thus, our experimental manipulations show that the dynamic state of individual cells can rapidly switch at multiple effective concentration levels of cytosolic GEF-H1. These observations directly confirmed that the GEF-H1 concentration is a critical parameter of this dynamic signal network. Our experimental tuning of the bifurcation parameter G_T directly validated the predictions of the numerical bifurcation analysis.

Influence of External Inputs into the Myosin Component on Rho Activity Dynamics

We noted that oscillations observed in the numerical simulations of the ODE model are always highly regular (Figure 2G). In contrast, experimentally observed oscillations were either regular (Figure 2G) or irregular (Figures 1A and 1B), suggesting that additional, extrinsic processes could influence the experimentally observed system dynamics. In particular, myosin activity dynamics are simplified in the ODE model, in which they are controlled only by Rho and a constant inhibitory baseline activity. However, in cells, myosin activity is known to be modulated by mechanical or biochemical inputs that originate from both extracellular and intracellular sources. For example, myosin activity is stimulated by viscoelastic counterforces (Luo et al., 2012), which originate from the mechanical properties of actomyosin itself and from associated subcellular structures, such as cell-substrate adhesions, the nucleus, or cell-cell junctions (Tojkander et al., 2012). In addition, these viscoelastic counterforces are modulated by stochastic fluctuations that originate from Brownian motion (Brangwynne et al., 2008), actin polymerization (Weber et al., 2015), adhesion clusters (Erdmann and Schwarz, 2004), and small actomyosin ensembles (Erdmann and Schwarz, 2012).

Thus, to derive a more realistic theoretical concept, we aimed to account for these complex and partially stochastic processes. To keep our theoretical concept as simple as possible, we considered a phenomenological description of these extracellular and intracellular inputs and modeled them by adding low levels of Gaussian noise to the myosin component to obtain a system of stochastic differential equations (SDEs) (see Equations 4, 5, and 6 in Method Details).

At low GEF-H1 concentrations, the SDE numerical simulations generated irregular, pulsatory dynamics (Figure 4A). At high GEF-H1 concentrations, the system showed no oscillations or pulses, but instead only low amplitude noise (Figure 4C). In contrast, at intermediate GEF-H1 concentrations, relatively regular, recurring pulses were observed (Figure 4B). To quantify peak irregularity, we measured the coefficient of variation (CV) of interpeak intervals. The application of this measure to SDE simulations predicted maximal peak regularity (i.e., lowest CV) at intermediate GEF-H1 concentrations (Figure 4D). To test this prediction, we analyzed interpeak intervals in cells that express varying levels of GEF-H1. Compared to simulations, the experimentally measured CV displayed relatively high cell-to-cell variability, suggesting that additional factors may influence the regularity of system dynamics. Nevertheless, as predicted by the simulations, the CV of interpeak intervals was reduced at intermediate expression levels (Figure 4E).

These results seem to suggest that the temporal dynamics of the GEF-H1/Rho/myosin signal network are relatively insensitive to extrinsic noise or other extrinsic inputs at intermediate and high cytosolic concentrations of GEF-H1. At intermediate levels,

CellPress
OPEN ACCESS

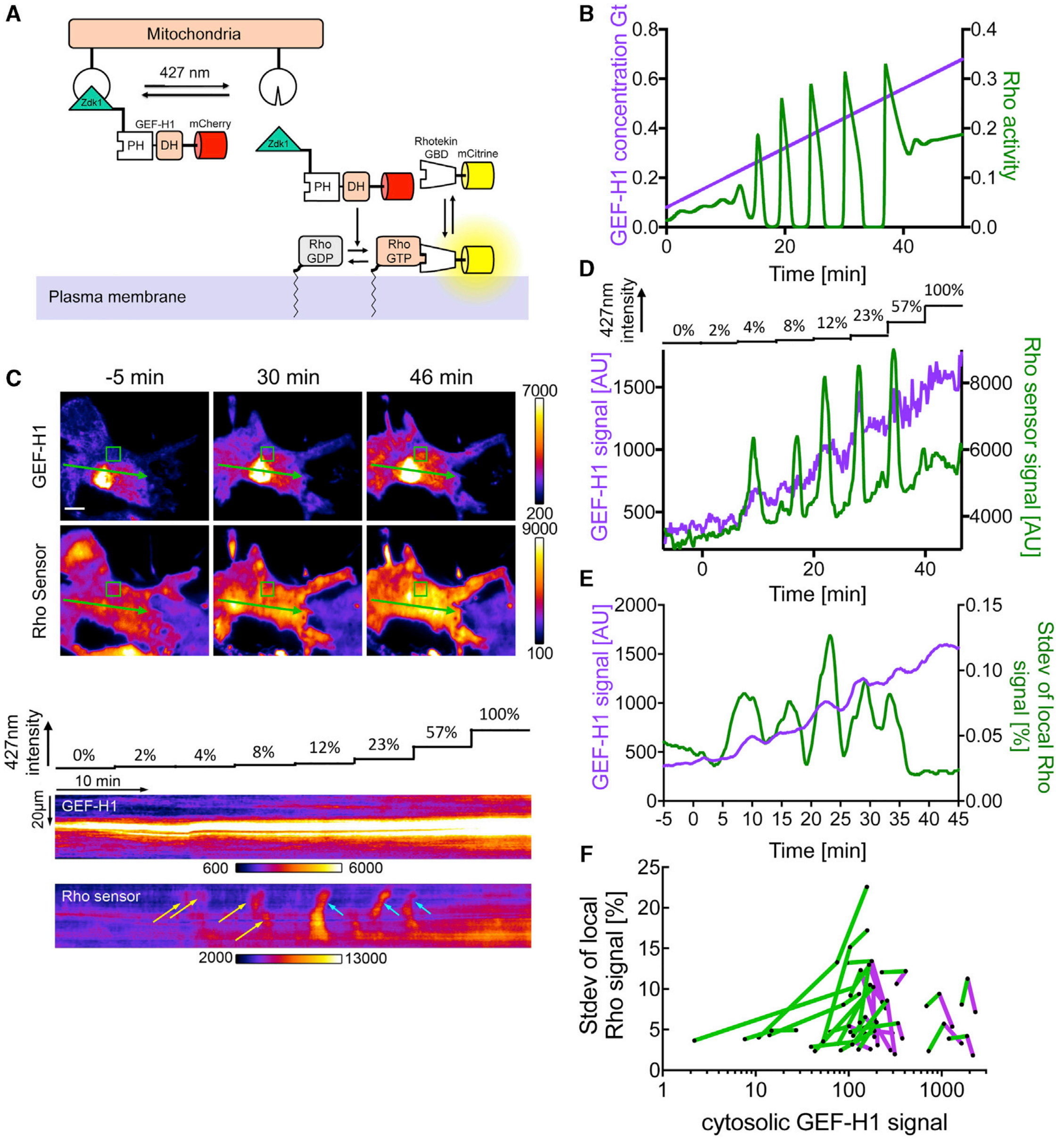

Figure 3. Model Prediction and Experimental Confirmation of GEF-H1 Concentration-Dependent Switching of Rho Activity Dynamics by Optogenetic Tuning

(A) A schematic representation of GEF-H1 release from mitochondria to tune the effective cytosolic GEF-H1 concentration with light.

(B) Simulation of Rho activity dynamics with linearly increasing total concentration of GEF-H1 (G_T).

(C–F) The dependence of Rho sensor activity dynamics on the effective, cytosolic GEF-H1 concentration was analyzed in U2OS cells expressing the LOV domain targeted to mitochondria (TOM20-LOV2), mCherry-Zdk1-GEF-H1(C53R), and a Rho sensor (mCitrine-Rhotekin-GBD).

(C–E) Analysis of a representative cell.

(C) Top: TIRF images of mCherry-Zdk1-GEF-H1(C53R) and the Rho sensor (see also Video S4). Bottom: kymographs corresponding to green arrows in top panel. Yellow arrows point to Rho activity pulses, cyan arrows point to Rho activity waves.

(D) Cytosolic mCherry-Zdk1-GEF-H1(C53R) levels obtained as the minimum signal in green boxed regions in (C), and measurements of the Rho activity sensor signal over the time course of the experiment.

(E) The local standard deviation of Rho activity signals over the time course of the experiment, using a shifting time-interval of 15 frames, average of all central cell regions of the cell shown in (C), and cytosolic mCherry-Zdk1-GEF-H1(C53R) levels (moving average of 15 frames corresponding to 2.5 min).

(F) Plot of local standard deviation of Rho activity signals against cytosolic mCherry-Zdk1-GEF-H1(C53R) levels from all of the analyzed cells (n = 23 cells from 3 experiments). Lines connect 3 data points that represent the following time intervals of optogenetic tuning experiments: the average of the initial 10 frames, the intermediate frames, and the last 10 frames. Lines with net increase of local Rho activity standard deviation are in green, lines with net decrease are in magenta.

(E and F) Percentage (%) indicates percentage of mean intensity.

Scale bars: 10 μm.

See also Figure S4.

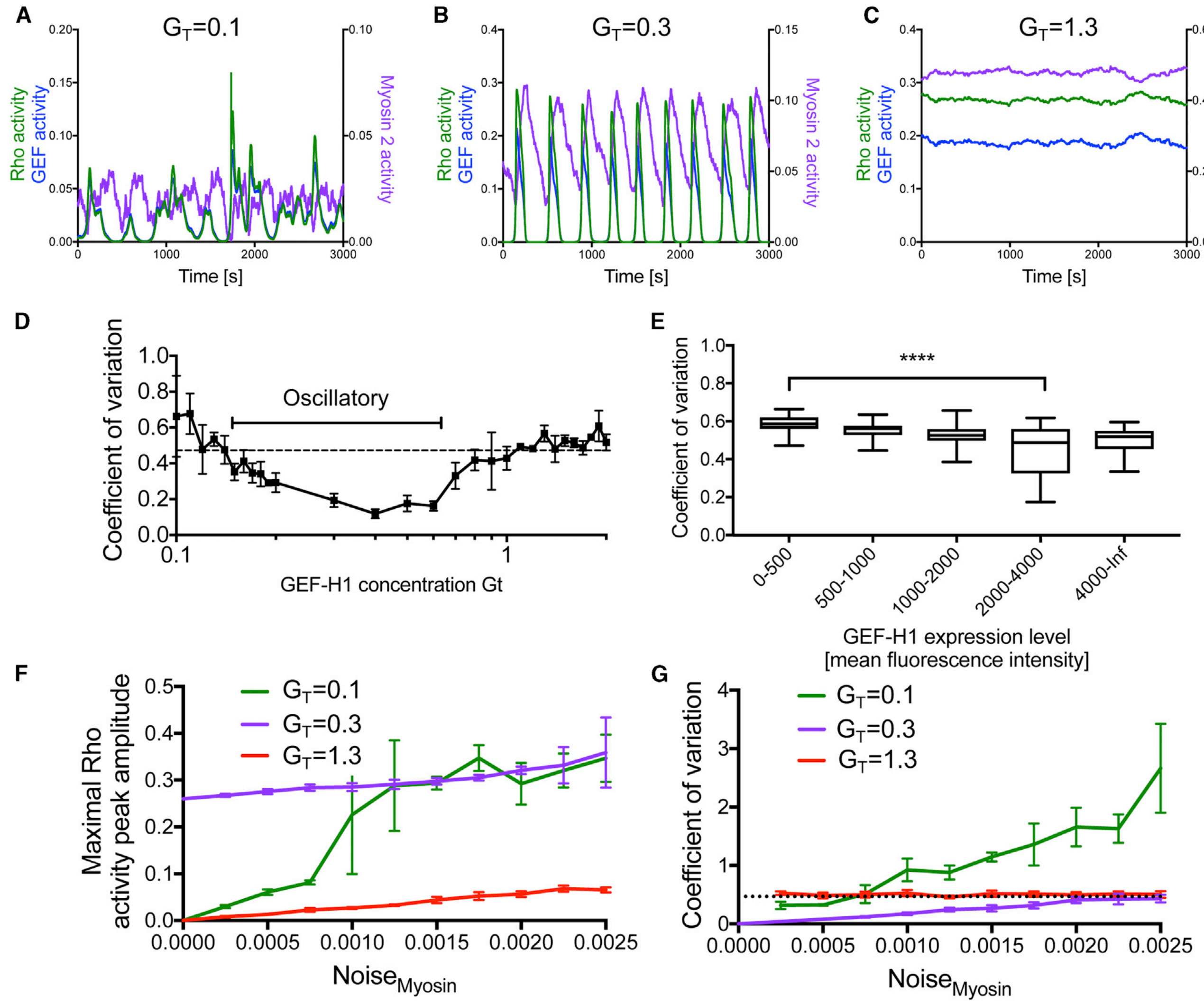

Figure 4. Sensitivity of Rho Activity Pulses to Stochastic Myosin Inputs

Numerical simulations of the system of stochastic differential equations (Equations 4, 5, and 6) that include extrinsic additive noise in the myosin component to represent extracellular and intracellular inputs.

(A–C) SDE simulations at low (A), intermediate (B), and high (C) cytosolic concentrations of GEF-H1 (G_T).

(D) Measurement of the coefficient of variation (CV) of the Rho activity interpeak distance in SDE simulations at various cytosolic concentrations of GEF-H1 (G_T). The black bar indicates the range of G_T that generates oscillatory dynamics in the absence of noise.

(E) Measurement of the CV of the Rho activity interpeak distance in experiments at varying expression levels of active, microtubule-binding deficient GEF-H1(C53R) mutant (n = 91 cells from 3 experiments; box and whisker plot; ****1-way ANOVA, Dunnett's post test, $p < 0.0001$).

(F) Dependence of maximal Rho activity peak amplitude on extrinsic noise levels in the myosin component ($Noise_{myosin}$) at low, intermediate, and high GEF-H1 concentrations (G_T).

(G) Dependence of the CV of the Rho activity interpeak distance on extrinsic noise levels in the myosin component at low, intermediate, and high GEF-H1 concentrations (G_T).

Dotted black line in (D) and (G): CV of Gaussian input noise. (D), (F), and (G): mean and SD from 3 independent simulations.

pulses do not need a trigger, leading to self-sustained oscillatory dynamics, and at high levels, a large fraction of the Rho component is constantly in the active form and activity dynamics are dampened. In contrast, at low cytosolic concentrations of GEF-H1, noise can trigger irregular pulses, suggesting that the system is most sensitive to extrinsic inputs under these conditions. To test this idea, we gradually increased the level of extrinsic noise in our SDE simulations and measured peak amplitude and peak regularity. We found that pulses with high maximal Rho activity amplitude are increasingly generated at low GEF-H1 concentrations with increasing noise (Figures 4A and 4F). At intermediate and high GEF-H1 concentrations, maximal Rho activity amplitude is largely independent of noise levels (Figures 4B, 4C, and 4F).

Peak irregularity, measured via the CV of interpeak intervals, increases most strongly with increasing noise levels at low

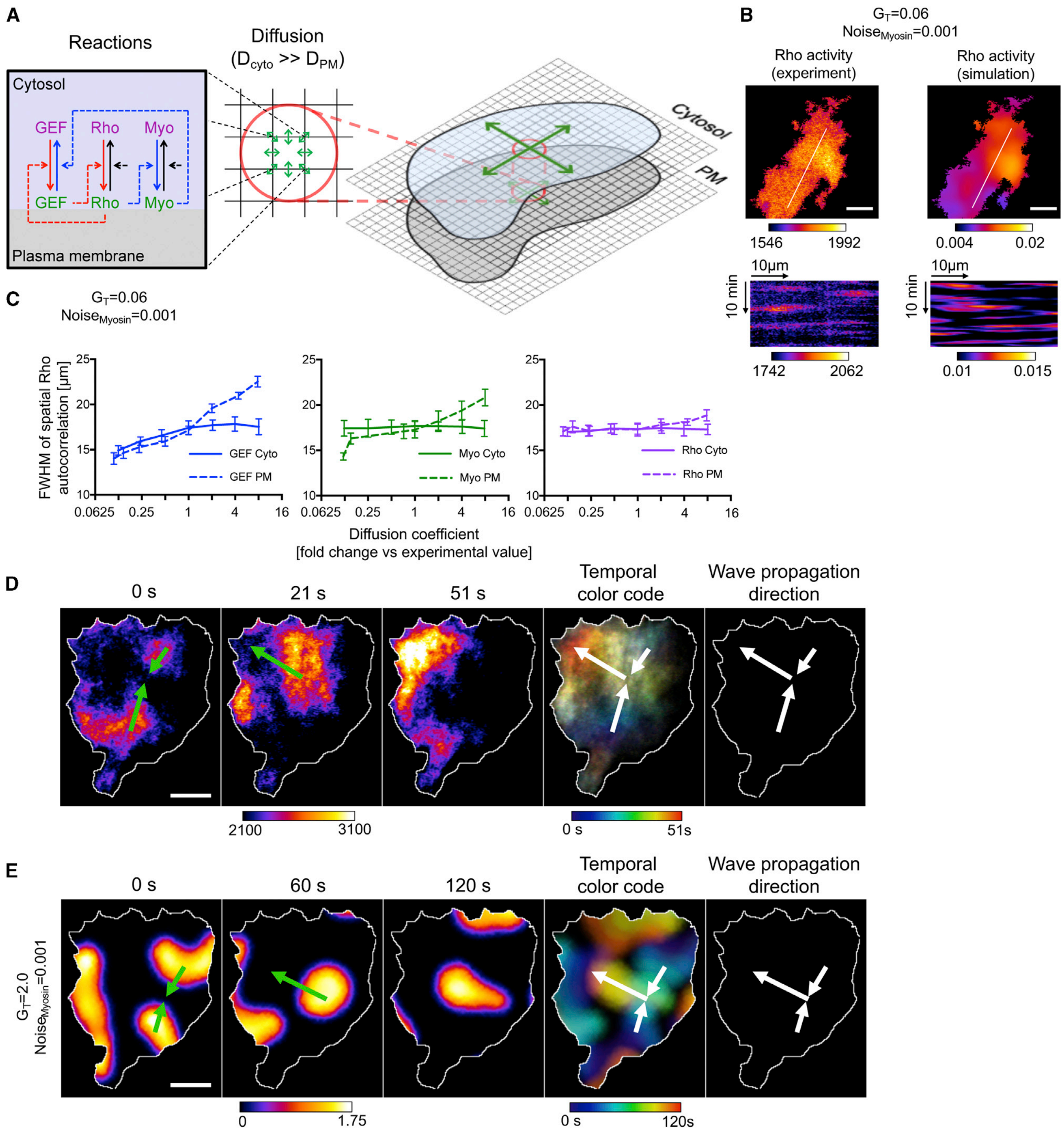

Figure 5. Spatial Patterning of Rho Activity Dynamics

(A) Schematic representation of the cellular automata model. Reactions (left) are simulated within individual discrete regions. Mass transfer between these regions is simulated based on fast and slow diffusive mobility of the cytosolic and plasma membrane-associated components (center). Cytosolic and plasma membrane regions of cells are represented by two 2-dimensional (2D) arrays of discrete spatial regions (right).

(B) Experimentally observed Rho activity sensor signals in U2OS cells obtained via TIRF microscopy (left panels) and numerical simulations of Rho activity obtained by using a cellular automata model (right panels). Top panels: representative time frame from experiments and simulations (see also Video S6); bottom panels: kymographs corresponding to white lines in top panels. Cells co-express the Rhotekin-GBD sensor and GEF-H1(C53R). The representative cell shown in this panel expresses GEF-H1(C53R) at low levels.

(C) Measurement of spatial Rho activity pulse width by fitting a 2D Gaussian to the spatial autocorrelation function of cellular automata simulations. Shown is the full width at half maximum (FWHM) of the Gaussian fit from simulations performed after multiplying diffusion coefficients with varying factors (mean and SD from 8 independent simulations).

(legend continued on next page)

GEF-H1 concentrations (Figure 4G). Thus, low total concentrations of the positive feedback mediator GEF-H1 are a prerequisite for triggering irregular pulses with high activity amplitude by stochastic inputs into the myosin component.

Spatial Patterning of Rho Activity Dynamics Is Dependent on Slow Diffusion of Membrane-Associated GEF-H1

In previous sections, local system dynamics were investigated, which revealed how pulsatile and oscillatory system dynamics can emerge from the positive and negative feedback regulation of Rho activity. However, to generate the observed spatiotemporal activity patterns, reactions must be coupled to transport processes such as diffusion (Meinhardt, 2004; Turing, 1952). We therefore extended the system of SDEs and performed numerical simulations using a cellular automata model (Figure 5A; Method Details) (Schmick et al., 2014). The reaction kinetic parameters that were obtained via the Bayesian fitting approach of the ODE system (Table S3) were used, and no additional parameter fitting was performed with the cellular automata model. Estimations for diffusion coefficients of the active, plasma membrane-associated and inactive, cytosolic system components were based on published data or experimental measurements (see Method Details). To represent external inputs, we used the lowest level of noise ($Noise_{myosin}$ = 0.001) that was capable of triggering stochastic Rho activity pulses when the system of SDEs was solved numerically (Figure 4).

At low cytosolic GEF-H1 concentrations, numerical simulations using this reaction-diffusion system (RDS, Equations 7, 8, 9, 10, 11, and 12) generated irregular, local Rho activity pulses that were very similar to corresponding experimental observations (Figure 5B; Video S6). This shows that coupling of the reactions of our temporal model with known diffusive mobility of the major system components is sufficient to generate spatiotemporal patterns that correspond to our experimental observations.

Pattern formation often depends on differential diffusive mobility of system components. Our RDS encompasses several components with distinct mobility, and their individual contribution to pattern formation is unclear. We therefore varied individual diffusion coefficients in a sensitivity analysis. This showed that up to 8-fold changes in diffusion coefficients did not prevent the formation of local Rho activity pulses per se; however, spatial pulse width was significantly altered (Figure 5C). The strongest dependence was observed when altering the diffusion coefficient of plasma membrane-associated GEF-H1.

In cells that express increased levels of active GEF-H1, we observed multiple, spatially focused wave structures that either annihilated each other or merged and changed their shape and propagation direction (Figure 5D; Video S7). We observed similar activity dynamics in our simulations if we increased the GEF-H1 concentration (Figure 5E). By adding spatial dimensions and analyzing the effect of coupling diffusion and reactions, we extended our theoretical framework to explain experimentally observed spatiotemporal patterns of cell contraction dynamics and predict that the diffusive mobility of the positive feedback mediator GEF-H1 is a critical parameter for activity pattern formation.

DISCUSSION

In this study, we introduced an improved method to acutely perturb and measure the activity state of signal network components and combined this experimental approach with theoretical analysis of the system dynamics. This strategy enabled us to infer causalities in a GEF-H1/Rho/myosin signal network and to derive a theoretical framework that can explain the formation of local pulses of Rho activity that control local cell contraction dynamics.

Rho Activity Amplification Mediated by Lbc-type RhoGEFs

An important insight of our study is that local Rho activity can be amplified via a very simple mechanism in which active Rho can recruit its own activator GEF-H1 to the plasma membrane. GEF-H1 shares this ability with other members of the Lbc family of RhoGEFs (Medina et al., 2013). Lbc-GEFs are conserved in vertebrate and invertebrate species and widely expressed in various mammalian cell types. Hence, these regulators may also play a role in other systems that generate local cell contraction pulses, such as during oocyte cytokinesis (Bement et al., 2015) or in tissue remodeling during embryogenesis (Coravos et al., 2017; Kasza and Zallen, 2011; Martin et al., 2009, 2010; Mason et al., 2016; Munjal et al., 2015; Nishikawa et al., 2017). In these systems, various additional mechanisms were proposed that may mediate positive feedback amplification in local cell contraction pulses (Bement et al., 2015; Munjal et al., 2015; Nishikawa et al., 2017; Wu, 2017). These alternative mechanisms are not mutually exclusive and could therefore act in parallel to Rho activity amplification via Lbc-GEFs to ensure robust generation of cell contraction pulses. Our study offers a simple mechanism and a small set of candidates that could play an important role in this process.

Simplifications in the Core Signal Network

In this study, we propose a very simple core reaction network that generates the observed signal network dynamics. The detailed, biochemical mechanisms that control Rho GTPases and myosin are much more complex and include solubilization by RhoGDIs, crosstalk between Rho family members, actin and myosin filament polymerization, and several myosin regulatory kinases and phosphatases. By simplifying these complex processes, our study suggests that the core reaction network captures two of the most important features of this system: the network topology, which includes coupled positive and negative feedback loops, and the kinetics of the reactions that mediate these feedback loops, which are fast and slow, respectively.

(D) Example for experimental observation of 2 focused wave fragments that fuse and change their shape and propagation direction. Cells co-express the mCherry-Rhotekin-GBD sensor and EGFP-GEF-H1. The representative cell shown in this panel expresses GEF-H1 at intermediate levels (see also Video S7).
(E) Cellular automata simulation of Rho activity dynamics shows similar activity patterns as shown in (D).
Scale bars: 10 μm.

Table 1. Comparison of the Estimated Values of the Michaelis Constants with Maximal Concentrations of Corresponding Theoretical System Components

Michaelis Constant, Mode, and 95% Credible Interval (in 10^6 Molecules/Cell)	Maximal Concentration of Corresponding System Component in Oscillatory State (in 10^6 Molecules/Cell)
Km1: 2.42 (0.0886–2.43)	inactive Rho: 0.44
Km2: 0.0745 (0.0741–0.564)	active Rho: 0.34
Km5: 0.014 (0.00733–0.0658)	inactive myosin: 1.21
Km6: 0.786 (0.258–1.83)	active myosin: 0.24

Maximal concentrations were obtained from the numerical analysis of the oscillatory state using Equations 1, 2, and 3 (see Method Details).

These core features are typical for oscillatory/excitable systems (Tyson et al., 2003) and enable rapid amplification of Rho at the onset of a pulse that is coupled to a slow, delayed self-inhibition that terminates this amplification phase.

The biochemical details of how these system features are implemented may be necessary to enable the large difference in the kinetics between the positive and negative feedback loops. Solubilization of inactive Rho by RhoGDI provides a large pool of cytosolic Rho that can be rapidly activated and transferred to the plasma membrane (Garcia-Mata et al., 2011). In contrast, polymerization of actin and myosin filaments is a relatively slow process that acts via many intermediate steps, which slows down the negative feedback loop.

The biochemical features of the individual Rho signal network components integrate the core network that generates oscillations with inputs that can modulate these dynamics and outputs that link to cellular functions. For example, the force that is generated by myosin is critical to alter cell shape and is involved in the transduction of mechanical signals, and the crosstalk between Rho and other regulators of cell morphodynamics, such as Rac, may play a role in coordinating protrusive and contractile cell shape changes.

The simplicity of the core network raises the question: can the mathematical description be simplified further? In particular, several reactions were implemented via saturable Michaelis-Menten kinetics, but if those occur far from saturation, then a simpler implementation via mass action may be sufficient. Comparing the values of the Michaelis constants with the maximal concentrations of the corresponding theoretical system components obtained from the bifurcation analysis (Table 1) suggests that the reactions that involve K_{m1} and K_{m6} occur far from saturation, as the values of these Michaelis constants are higher than the corresponding concentrations of inactive Rho and active myosin. The upper limit of the 95% credible region of K_{m2} is also relatively high compared to the corresponding concentration of active Rho, suggesting that this also applies to some extent to K_{m2}. This is in contrast to K_{m5}, which is much lower than the corresponding concentration of inactive myosin. Thus, saturation and the associated non-linearity in the Michaelis-Menten terms is predominantly relevant for the reaction involving K_{m5}. The corresponding reaction is indeed expected to be highly non-linear, as it represents a multi-step enzymatic cascade that involves several kinases, phosphatases, and actin polymerization (Riento and Ridley, 2003).

The parameters that we obtained by constraining our simplified core system with experimental data allows further insight into system dynamics. For example, the observed local dynamics of active Rho and myosin at the plasma membrane occur in the range of minutes; however, these timescales are likely dominated by the relatively slow, rate-limiting reactions in the negative feedback. Our parameter estimation suggests that the turnover of active Rho molecules at the plasma membrane is much faster (estimated dwell time of active RhoA; k_2/Km_2 ~0.5 s) than the observed local dynamics of active Rho.

Emergence of Spatiotemporal Cell Contraction Patterns

Both in our experiments and in our reaction-diffusion simulations, we observed focused pulses and wave structures with increased Rho activity. Interestingly, these patterns are similar to those predicted for three-component RDSs in the influential paper by Alan Turing on the chemical basis of morphogenesis (Turing, 1952), which were subsequently studied in more detail by Hans Meinhardt (Meinhardt, 2004). In such systems, a single activator is regulated by two distinct inhibitors, one local, long-lasting inhibitor that controls oscillations in time, and one long-ranging inhibitor that controls patterns in space (Meinhardt, 2004). These requirements are met in our experimental system: active myosin acts as a slow, long-lasting local inhibitor that controls oscillatory activity dynamics. Depletion of the activator by diffusion of inactive GEF-H1 enables long-range inhibition to generate patterns of cell contraction pulses in space (Figure 6A).

In the regime in which local pulses formed, GEF-H1 was present at low concentrations and thus present in limiting amounts (~21% active fraction at steady-state in simulation shown in Figure 5B), which is a prerequisite for the depletion-based long-range negative feedback (Figure 6A). In this low GEF-H1 concentration range, Rho was not limiting (~2.2% active fraction in simulations), which can explain why the diffusion of GEF-H1 is more critical for pattern formation than Rho. Myosin was also not present in limiting amounts (~0.9% active fraction in simulations); however, slow myosin diffusion is necessary to maintain its function as a slow, long-lasting, short-range inhibitor.

Rho activity waves often appeared as moving spots or wavefront fragments in both experiments and simulations (Figures 5B, 5D, and 5E). This is in contrast to symmetric, radial wave propagation (Figure 6B), which is observed in simpler excitable systems such as the Belousov-Zhabotinsky reaction (Belousov, 1959; Zhabotinsky, 1964) that include short-range positive feedback and short-range negative feedback without the long-range negative feedback component. The patterns that we observe are more similar to those that were described by Meinhardt for three-component RDSs (Meinhardt, 2004).

Sensitivity of Cell Contraction Signal Network Dynamics to External Inputs

We previously reported that contractility pulses in U2OS cells are modulated by the elasticity of the extracellular matrix, suggesting that they participate in a mechanotransduction process (Graessl et al., 2017). Here, we used extrinsic stochastic inputs into the myosin component as a phenomenological

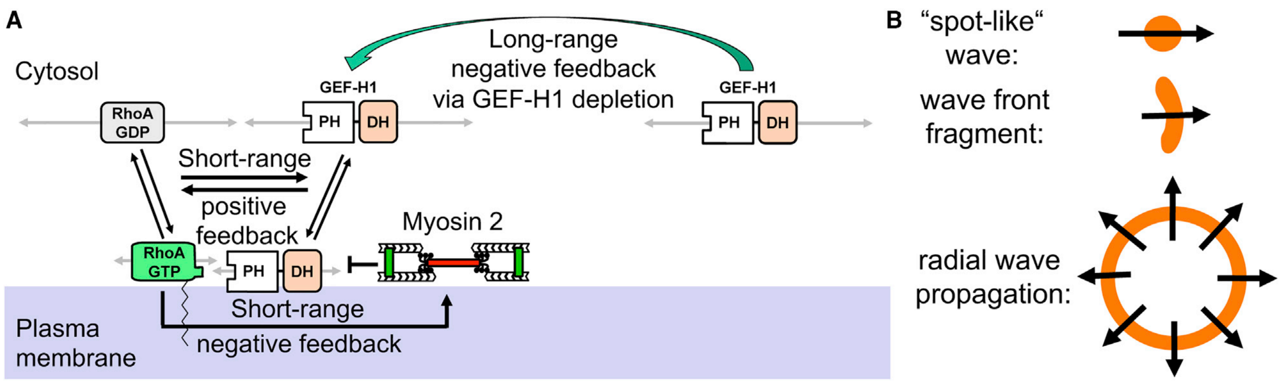

Figure 6. Proposed Pattern Formation Mechanism That Generates Rho Activity Pulses and Waves

(A) Slow-diffusing active Rho and myosin near the plasma membrane and fast-diffusing inactive components in the cytosol form a reaction-diffusion system (RDS; Equations 7, 8, 9, 10, 11, and 12) that includes short-range positive and negative feedback, as well as a long-range negative feedback via substrate depletion (green arrow). Gray arrows: diffusion; black arrows: translocations and reactions.

(B) Schematic representation of propagating focused spot-like waves and focused wave fragments, as opposed to radial wave propagation, which does not require a long-range spatial focusing mechanism.

approximation of biochemical or mechanical input signals. We find that the regular activity pulses that are induced by intermediate GEF-H1 concentrations are insensitive to such external inputs and thus unlikely to be directly modulated in this state. In contrast, at low concentrations of GEF-H1 that correspond to its endogenous levels, external inputs can trigger focused Rho activity pulses. This insight has important implications for the potential role of Lbc-type GEFs in cell contraction signaling. While low levels of active Lbc GEFs in unperturbed U2OS cells may play a role in sensing external inputs, higher levels of active Lbc GEFs may play a role in driving cell-autonomous contraction oscillations that are observed during embryonic development.

Our study thus provides a theoretical framework for cell contraction pattern formation and its modulation by biochemical and mechanical signals. Our quantitative analysis of this minimal system that operates within subcellular areas in individual cells provides a basis to understand how this system is interlinked with cellular function and may thereby also help to improve our understanding of cell contraction patterns that are observed in more complex multicellular systems.

STAR★METHODS

Detailed methods are provided in the online version of this paper and include the following:

- KEY RESOURCES TABLE
- RESOURCE AVAILABILITY
  - Lead contact
  - Materials availability
  - Data and code availability
- EXPERIMENTAL MODEL AND SUBJECT DETAILS
  - Cell culture
- METHOD DETAILS
  - Transfection and pharmocological treatments
  - Plasmid construction
  - Microscopy and chemo-optogenetics
  - Simplified molecular activity painting
  - Optogenetic tuning of cytosolic GEF-H1
  - Theoretical methods
  - Bifurcation analysis
  - Simulation of the SDE system
  - Cellular automata simulations
- QUANTIFICATION AND STATISTICAL ANALYSIS
  - Analysis of signal network dynamics in cells
  - Analysis of dynamics in simulations
  - Statistical Analysis

SUPPLEMENTAL INFORMATION

Supplemental Information can be found online at https://doi.org/10.1016/j.celrep.2020.108467.

ACKNOWLEDGMENTS

We thank Sven Müller (MPI Dortmund) for expert microscopy support, Malte Schmick and Aneta Koseska for helpful discussions, Jessica Wagner for the generation of the optimized LOVTRAP construct, Stefanie Gossen for contributing to perturbation experiments, and Philippe Bastiaens (MPI Dortmund) for departmental support and helpful discussions. This work was supported by the DFG Heisenberg Program (grant no. DE 823/3-1, DE 823/4-1, DE 823/6-1 and DE 823/8-1) to L.D. and D.K.; MERCUR grant no. Pr-2012-0022 to L.D. and P.N.; FORSYS partner initiative (BMBF grant no. 0315258) to L.D.; DFG Priority Programme SPP 1462/2 to P.N.; and the Marie Skłodowska-Curie Innovative Training Network InCeM (European Union, Call: H2020-MSCA-ITN-2014, Project ID: 642866) to V.O.J., S.P., A.M., L.D., and P.N. A.M., S.P., L.D., and P.N. thank the Isaac Newton Institute for Mathematical Sciences for its hospitality during the program Coupling Geometric PDEs with Physics for Cell Morphology, Motility and Pattern Formation (EPSRC EP/K032208/1). A.M. and E.C.-F. acknowledge support from the Leverhulme Trust Project Grant (RPG-2014-149). A.M. is a Royal Society Wolfson Research Merit Award Holder funded generously by the Wolfson Foundation. S.P. is supported by Natural Sciences Engineering Research Council Discovery grant (RGPIN-2018-04967). Y.W.W. is thankful for the support from the ERC (ChemBioAP), Vetenskapsrådet (no. 2018-04585), and The Knut and Alice Wallenberg Foundation.

AUTHOR CONTRIBUTIONS

Conceptualization, L.D., P.N., A.M., and S.P.; Methodology, L.D., S.P., A.M., D.K., V.O.J., and E.C.-F.; Software, V.O.J., E.C.-F., L.D., A.M., S.P., S.B., and T.M.; Formal Analysis, V.O.J., L.D., E.C.-F., S.P., and A.M.; Investigation, D.K., J.K., and M.G.; Resources, X.C. and Y.-W.W.; Writing—Original Draft, L.D., P.N., S.P., and A.M.; Writing—Review and Editing, D.K., V.O.J., and E.C.-F.; Visualization, L.D., D.K., and P.N.; Supervision, L.D., A.M., S.P., and P.N.; Funding Acquisition, L.D., P.N., A.M., S.P., and Y.-W.W.

DECLARATION OF INTERESTS

The authors declare no competing interests.

Received: May 7, 2020
Revised: September 16, 2020
Accepted: November 10, 2020
Published: December 1, 2020

REFERENCES

Baird, M.A., Billington, N., Wang, A., Adelstein, R.S., Sellers, J.R., Fischer, R.S., and Waterman, C.M. (2017). Local pulsatile contractions are an intrinsic property of the myosin 2A motor in the cortical cytoskeleton of adherent cells. Mol. Biol. Cell *28*, 240–251.

Beck, M., Schmidt, A., Malmstroem, J., Claassen, M., Ori, A., Szymborska, A., Herzog, F., Rinner, O., Ellenberg, J., and Aebersold, R. (2011). The quantitative proteome of a human cell line. Mol. Syst. Biol. *7*, 549.

Belousov, B.P. (1959). Цериодически действующая реакция и ее мечанизм [Periodically acting reaction and its mechanism]. In Сборник рефератов Цо радиационной медицине [Collection of Abstracts on Radiation Medicine], R.J. Field and M. Burger, eds. (Medgiz), pp. 145–147.

Bement, W.M., Leda, M., Moe, A.M., Kita, A.M., Larson, M.E., Golding, A.E., Pfeuti, C., Su, K.C., Miller, A.L., Goryachev, A.B., and von Dassow, G. (2015). Activator-inhibitor coupling between Rho signalling and actin assembly makes the cell cortex an excitable medium. Nat. Cell Biol. *17*, 1471–1483.

Brangwynne, C.P., Koenderink, G.H., MacKintosh, F.C., and Weitz, D.A. (2008). Cytoplasmic diffusion: molecular motors mix it up. J. Cell Biol. *183*, 583–587.

Campillo-Funollet, E., Venkataraman, C., and Madzvamuse, A. (2019). Bayesian Parameter Identification for Turing Systems on Stationary and Evolving Domains. Bull. Math. Biol. *81*, 81–104.

Chang, Y.C., Nalbant, P., Birkenfeld, J., Chang, Z.F., and Bokoch, G.M. (2008). GEF-H1 couples nocodazole-induced microtubule disassembly to cell contractility via RhoA. Mol. Biol. Cell *19*, 2147–2153.

Chen, X., Venkatachalapathy, M., Kamps, D., Weigel, S., Kumar, R., Orlich, M., Garrecht, R., Hirtz, M., Niemeyer, C.M., Wu, Y.W., and Dehmelt, L. (2017). "Molecular Activity Painting": Switch-like, Light-Controlled Perturbations inside Living Cells. Angew. Chem. Int. Ed. Engl. *56*, 5916–5920.

Citi, S., and Kendrick-Jones, J. (1987). Regulation of non-muscle myosin structure and function. BioEssays *7*, 155–159.

Coravos, J.S., Mason, F.M., and Martin, A.C. (2017). Actomyosin Pulsing in Tissue Integrity Maintenance during Morphogenesis. Trends Cell Biol. *27*, 276–283.

Costantini, L.M., Baloban, M., Markwardt, M.L., Rizzo, M., Guo, F., Verkhusha, V.V., and Snapp, E.L. (2015). A palette of fluorescent proteins optimized for diverse cellular environments. Nat. Commun. *6*, 7670.

Cui, Y., Hameed, F.M., Yang, B., Lee, K., Pan, C.Q., Park, S., and Sheetz, M. (2015). Cyclic stretching of soft substrates induces spreading and growth. Nat. Commun. *6*, 6333.

Dulyaninova, N.G., House, R.P., Betapudi, V., and Bresnick, A.R. (2007). Myosin-IIA heavy-chain phosphorylation regulates the motility of MDA-MB-231 carcinoma cells. Mol. Biol. Cell *18*, 3144–3155.

Engler, A.J., Sen, S., Sweeney, H.L., and Discher, D.E. (2006). Matrix elasticity directs stem cell lineage specification. Cell *126*, 677–689.

Erdmann, T., and Schwarz, U.S. (2004). Stochastic dynamics of adhesion clusters under shared constant force and with rebinding. J. Chem. Phys. *121*, 8997–9017.

Erdmann, T., and Schwarz, U.S. (2012). Stochastic force generation by small ensembles of myosin II motors. Phys. Rev. Lett. *108*, 188101.

Garcia-Mata, R., Boulter, E., and Burridge, K. (2011). The 'invisible hand': regulation of RHO GTPases by RHOGDIs. Nat. Rev. Mol. Cell Biol. *12*, 493–504.

Goedhart, J., von Stetten, D., Noirclerc-Savoye, M., Lelimousin, M., Joosen, L., Hink, M.A., van Weeren, L., Gadella, T.W., Jr., and Royant, A. (2012). Structure-guided evolution of cyan fluorescent proteins towards a quantum yield of 93%. Nat. Commun. *3*, 751.

Gorfinkiel, N., and Blanchard, G.B. (2011). Dynamics of actomyosin contractile activity during epithelial morphogenesis. Curr. Opin. Cell Biol. *23*, 531–539.

Graessl, M., Koch, J., Calderon, A., Kamps, D., Banerjee, S., Mazel, T., Schulze, N., Jungkurth, J.K., Patwardhan, R., Solouk, D., et al. (2017). An excitable Rho GTPase signaling network generates dynamic subcellular contraction patterns. J. Cell Biol. *216*, 4271–4285.

Griesbeck, O., Baird, G.S., Campbell, R.E., Zacharias, D.A., and Tsien, R.Y. (2001). Reducing the environmental sensitivity of yellow fluorescent protein. Mechanism and applications. J. Biol. Chem. *276*, 29188–29194.

Hodge, R.G., and Ridley, A.J. (2016). Regulating Rho GTPases and their regulators. Nat. Rev. Mol. Cell Biol. *17*, 496–510.

Kasza, K.E., and Zallen, J.A. (2011). Dynamics and regulation of contractile actin-myosin networks in morphogenesis. Curr. Opin. Cell Biol. *23*, 30–38.

Kim, E.J.Y., Korotkevich, E., and Hiiragi, T. (2018). Coordination of Cell Polarity, Mechanics and Fate in Tissue Self-organization. Trends Cell Biol. *28*, 541–550.

Krendel, M., Zenke, F.T., and Bokoch, G.M. (2002). Nucleotide exchange factor GEF-H1 mediates cross-talk between microtubules and the actin cytoskeleton. Nat. Cell Biol. *4*, 294–301.

Lee, C.S., Choi, C.K., Shin, E.Y., Schwartz, M.A., and Kim, E.G. (2010). Myosin II directly binds and inhibits Dbl family guanine nucleotide exchange factors: a possible link to Rho family GTPases. J. Cell Biol. *190*, 663–674.

Liu, P., Calderon, A., Konstantinidis, G., Hou, J., Voss, S., Chen, X., Li, F., Banerjee, S., Hoffmann, J.E., Theiss, C., et al. (2014). A bioorthogonal small-molecule-switch system for controlling protein function in live cells. Angew. Chem. Int. Ed. Engl. *53*, 10049–10055.

Luo, T., Mohan, K., Srivastava, V., Ren, Y., Iglesias, P.A., and Robinson, D.N. (2012). Understanding the cooperative interaction between myosin II and actin cross-linkers mediated by actin filaments during mechanosensation. Biophys. J. *102*, 238–247.

Maître, J.L., Niwayama, R., Turlier, H., Nédélec, F., and Hiiragi, T. (2015). Pulsatile cell-autonomous contractility drives compaction in the mouse embryo. Nat. Cell Biol. *17*, 849–855.

Martin, A.C., Kaschube, M., and Wieschaus, E.F. (2009). Pulsed contractions of an actin-myosin network drive apical constriction. Nature *457*, 495–499.

Martin, A.C., Gelbart, M., Fernandez-Gonzalez, R., Kaschube, M., and Wieschaus, E.F. (2010). Integration of contractile forces during tissue invagination. J. Cell Biol. *188*, 735–749.

Mason, F.M., Xie, S., Vasquez, C.G., Tworoger, M., and Martin, A.C. (2016). RhoA GTPase inhibition organizes contraction during epithelial morphogenesis. J. Cell Biol. *214*, 603–617.

Medina, F., Carter, A.M., Dada, O., Gutowski, S., Hadas, J., Chen, Z., and Sternweis, P.C. (2013). Activated RhoA is a positive feedback regulator of the Lbc family of Rho guanine nucleotide exchange factor proteins. J. Biol. Chem. *288*, 11325–11333.

Meinhardt, H. (2004). Out-of-phase oscillations and traveling waves with unusual properties: the use of three-component systems in biology. Physica D *199*, 264–277.

Munjal, A., Philippe, J.M., Munro, E., and Lecuit, T. (2015). A self-organized biomechanical network drives shape changes during tissue morphogenesis. Nature *524*, 351–355.

Nalbant, P., and Dehmelt, L. (2018). Exploratory cell dynamics: a sense of touch for cells? Biol. Chem. *399*, 809–819.

Nishikawa, M., Naganathan, S.R., Julicher, F., and Grill, S.W. (2017). Controlling contractile instabilities in the actomyosin cortex. eLife *6*, e19595.

Petrásek, Z., Hoege, C., Mashaghi, A., Ohrt, T., Hyman, A.A., and Schwille, P. (2008). Characterization of protein dynamics in asymmetric cell division by scanning fluorescence correlation spectroscopy. Biophys. J. *95*, 5476–5486.

Platen, E. (1999). An introduction to numerical methods for stochastic differential equations. Acta Numer. *8*, 197–246.

Plotnikov, S.V., Pasapera, A.M., Sabass, B., and Waterman, C.M. (2012). Force fluctuations within focal adhesions mediate ECM-rigidity sensing to guide directed cell migration. Cell *151*, 1513–1527.

Riento, K., and Ridley, A.J. (2003). Rocks: multifunctional kinases in cell behaviour. Nat. Rev. Mol. Cell Biol. *4*, 446–456.

Rossman, K.L., Der, C.J., and Sondek, J. (2005). GEF means go: turning on RHO GTPases with guanine nucleotide-exchange factors. Nat. Rev. Mol. Cell Biol. *6*, 167–180.

Saha, S., Nagy, T.L., and Weiner, O.D. (2018). Joining forces: crosstalk between biochemical signalling and physical forces orchestrates cellular polarity and dynamics. Philos. Trans. R. Soc. Lond. B Biol. Sci. *373*, 20170145.

Schindelin, J., Arganda-Carreras, I., Frise, E., Kaynig, V., Longair, M., Pietzsch, T., Preibisch, S., Rueden, C., Saalfeld, S., Schmid, B., et al. (2012). Fiji: an open-source platform for biological-image analysis. Nat. Methods *9*, 676–682.

Schmick, M., Vartak, N., Papke, B., Kovacevic, M., Truxius, D.C., Rossmannek, L., and Bastiaens, P.I.H. (2014). KRas localizes to the plasma membrane by spatial cycles of solubilization, trapping and vesicular transport. Cell *157*, 459–471.

Schneider, C.A., Rasband, W.S., and Eliceiri, K.W. (2012). NIH Image to ImageJ: 25 years of image analysis. Nat. Methods *9*, 671–675.

Subauste, M.C., Von Herrath, M., Benard, V., Chamberlain, C.E., Chuang, T.H., Chu, K., Bokoch, G.M., and Hahn, K.M. (2000). Rho family proteins modulate rapid apoptosis induced by cytotoxic T lymphocytes and Fas. J. Biol. Chem. *275*, 9725–9733.

Tojkander, S., Gateva, G., and Lappalainen, P. (2012). Actin stress fibers–assembly, dynamics and biological roles. J. Cell Sci. *125*, 1855–1864.

Turing, A.M. (1952). The Chemical Basis of Morphogenesis. Philos. Trans. R. Soc. Lond. B Biol. Sci. *237*, 37–72.

Turkmann, M.A.A., Paulino, C.D., and Müller, P. (2019). Computational Bayesian Statistics: An Introduction*Volume 11* (Cambridge University Press).

Tyson, J.J., Chen, K.C., and Novak, B. (2003). Sniffers, buzzers, toggles and blinkers: dynamics of regulatory and signaling pathways in the cell. Curr. Opin. Cell Biol. *15*, 221–231.

Wang, H., Vilela, M., Winkler, A., Tarnawski, M., Schlichting, I., Yumerefendi, H., Kuhlman, B., Liu, R., Danuser, G., and Hahn, K.M. (2016). LOVTRAP: an optogenetic system for photoinduced protein dissociation. Nat. Methods *13*, 755–758.

Weber, C.A., Suzuki, R., Schaller, V., Aranson, I.S., Bausch, A.R., and Frey, E. (2015). Random bursts determine dynamics of active filaments. Proc. Natl. Acad. Sci. USA *112*, 10703–10707.

Wei, Q., and Adelstein, R.S. (2000). Conditional expression of a truncated fragment of nonmuscle myosin II-A alters cell shape but not cytokinesis in HeLa cells. Mol. Biol. Cell *11*, 3617–3627.

Weitzman, M.D. (2013). Single Molecule Dynamics of the Rho GTPase Spatial Cycle in Living Cells (Proquest), p. 164.

Wu, M. (2017). Pulses and waves of contractility. J. Cell Biol. *216*, 3899–3901.

Zhabotinsky, A.M. (1964). Periodic course of oxidation of malonic acid in solution (study of the Belousov reaction kinetics). Biofizika *9*, 306–311.

CellPress
OPEN ACCESS

STAR★METHODS

KEY RESOURCES TABLE

REAGENT or RESOURCE	SOURCE	IDENTIFIER
Antibodies		
Anti-VSV-G tag antibody	Abcam	Cat#ab34774; RRID: AB_778903
Bacterial and Virus Strains		
XL10-Gold® Ultracompetent Cells	Agilent Technologies, Inc	Cat#200314
Chemicals, Peptides, and Recombinant Proteins		
Collagen from calf skin	Sigma-Aldrich	Cat#C8919; CAS: 9007-34-5
EZ-Link Sulfo-NHS-Biotin	Thermo Scientific	Cat#21217; CAS: 119616-38-5
Nocodazole	Sigma-Aldrich	Cat#M1404; CAS: 31430-18-9
Nvoc-TMP-Cl photocaged chemical dimerizer	Chen et al., 2017	N/A
Poly-L-lysine-hydrobromide	Sigma-Aldrich	Cat#1274; CAS: 25988-63-0
Streptavidin	Serva	Cat#35490.02; CAS: 9013-20-1
Streptavidin Alexa Fluor 750	Life Technologies	Cat#S-21384
Experimental Models: Cell Lines		
Human U2OS osteosarcoma cells	ATCC	ATCC HTB-96; RRID: CVCL_0042
Recombinant DNA		
delCMV-mCitrine-Rhotekin-GBD	This paper	N/A
mCherry-GEF-H1 C53R	This paper	N/A
mCherry-GEF-H1 PH	This paper	N/A
mCitrine-GEF-H1 PH F539A-I541E	This paper	N/A
mTurquoise2-NES-eDHFR-RhoA Q63L ΔCAAX	This paper	N/A
mTurquoise2-NES-eDHFR-RhoA F30L ΔCAAX	This paper	N/A
mTurquoise2-NES-eDHFR-RhoA wt ΔCAAX	This paper	N/A
mTurquoise2-NES-eDHFR-RhoA T19N ΔCAAX	This paper	N/A
mCherry-Zdk1-GEF-H1 C53R	This paper	N/A
mTurquoise2-eDHFR-GEF-H1 (C53R)	This paper	N/A
mTurquoise2-eDHFR-LARG	This paper	N/A
pTriEx-NTOM20-CCL-moxBFP-CCL-LOV2	This paper	N/A
delCMV-mCherry-Rhotekin-GBD	Graessl et al., 2017	N/A
eDHFR-mCitrine	Chen et al., 2017	N/A
TagBFP-Halotag-CAAX	Chen et al., 2017	N/A
VSVG HaloTag-PARC	Chen et al., 2017	N/A
pTriEx-NTOM20-LOV2	Wang et al., 2016	Addgene Plasmid #81009
pTriEx-mCherry-Zdk1	Wang et al., 2016	Addgene Plasmid #81057
pTriEx-NTOM20-mVenus-LOV2	Wang et al., 2016; Kind gift of Klaus Hahn	N/A
mCitrine-N1	Griesbeck et al., 2001	Addgene Plasmid #54594
mCherry-C1	Clontech	Cat# 632524
moxBFP	Costantini et al., 2015	Addgene Plasmid #68064
EGFP-NMHCIIA	Wei and Adelstein, 2000	Addgene Plasmid #11347
mCherry-NMHCIIA	Dulyaninova et al., 2007	Addgene Plasmid #35687

(Continued on next page)

Continued

REAGENT or RESOURCE	SOURCE	IDENTIFIER
EGFP-2xFKPB'-Rac1 Q61L ΔCAAX	Liu et al., 2014	N/A
mCherry-LARG	Kind gift of Oliver Rocks	N/A
EGFP-GEF-H1 C53R	Krendel et al., 2002	N/A
pmTurquoise2-NES	Goedhart et al., 2012	Addgene Plasmid #36206
pcDNA3-EGFP-RhoAQ63L	Subauste et al., 2000; Kind gift of Gary Bokoch	Addgene Plasmid #12968
Software and Algorithms		
MATLAB R2018	MathWorks	https://de.mathworks.com
ImageJ	Schneider et al., 2012	https://imagej.nih.gov/ij/
Fiji	Schindelin et al., 2012	https://imagej.net/Fiji
Graphpad Prism 7.04 for Windows	GraphPad Software, Inc	https://www.graphpad.com
XPPauto 7.0		http://www.math.pitt.edu/~bard/xpp/xpp.html
Parameter fitting and posterior distribution analysis scripts	This paper	GitHub;https://github.com/ecam85/optogenetics
custom code for bifurcation analysis, ODE, SDE and CA simulations	This paper	GitHub;https://github.com/agdehmelt/optogenetic_tuning
Cellular automata algorithm	Schmick et al., 2014	N/A

RESOURCE AVAILABILITY

Lead contact

Further information and requests for resources and reagents should be directed to and will be fulfilled by the Lead Contact, Leif Dehmelt (leif.dehmelt@mpi-dortmund.mpg.de).

Materials availability

Plasmids generated in this study are available from the lead contact upon request.

Data and code availability

The custom code for parameter fitting, posterior distribution analysis, bifurcation analysis, ODE, SDE and CA simulations is available at: https://github.com/ecam85/optogenetics and https://github.com/agdehmelt/optogenetic_tuning.

EXPERIMENTAL MODEL AND SUBJECT DETAILS

Cell culture

Human U2OS osteosarcoma cells (ATCC HTB-96, RRID:CVCL_0042, female) were cultured in DMEM-GlutaMAX (GIBCO) supplemented with 10% fetal bovine serum (Pan Biotech), 50 U/ml penicillin, and 50 μg/ml streptomycin (Pan Biotech) at 37°C and 5% CO_2. For live-cell imaging, cells were plated onto collagen type I (Sigma-Aldrich) coated glass bottom dishes (1 h, 37°C, c = 0.01 mg/ml).

METHOD DETAILS

Transfection and pharmacological treatments

All plasmid DNA was transfected using Lipofectamine2000. To stimulate RhoA activity, U2OS cells were treated with the microtubule depolymerizing compound nocodazole (Sigma-Aldrich) (30 μM, 45–90 min). Pharmacological treatments started 14-20 h after transfection of plasmid DNAs.

Plasmid construction

The Rho activity sensor delCMV-mCherry-Rhotekin-GBD was described previously (Graessl et al., 2017). delCMV-mCitrine-Rhotekin-GBD was generated by replacing mCherry from delCMV-mCherry-Rhotekin-GBD with mCitrine from mCitrine-N1 (Clontech) using NheI and BsrGI restriction sites. EGFP-GEF-H1 C53R was described previously (Krendel et al., 2002). mCherry-GEF-H1 C53R was generated by replacing EGFP with mCherry from mCherry-C1 (Clontech) using NdeI and KpnI restriction sites. mCherry-GEF-H1 PH was generated using Gibson assembly. First, the GEF-H1 PH domain (AA 438-576) was amplified via PCR adding an

N-terminal GSGSGS linker with primers 5′-accggcggcatggacgagctgtacaagggttctggaagtggatccGGGGCCCGTCTGCAGGAG-3′ and 5′-ccgtcgactgcagaattcgatcaCTCCCTGGATGGGCATGTG-3′, followed by Gibson assembly with mCherry-C1 (Clontech) that was cut using BsrGI and HindIII restriction sites. mCherry-GEF-H1 PH F539A-I541E was generated by site-directed mutagenesis via the QuikChange protocol using the primers 5′-CCAGGAGAAAGGGATGGCACTGGAAAGCGCAGCCCCACCTGAG-3′ and 5′-GGTGGGGCTGCGCTTTCCAGTGCCATCCCTTTCTCCTGGTTGG-3′. mTurquoise2-NES-eDHFR-RhoA Q63L ΔCAAX (abbreviated as eDHFR-Rho Q63L) was derived from EGFP-2xFKPB'-Rac1 Q61L ΔCAAX (Liu et al., 2014) by first exchanging EGFP for mTurquoise2-NES (from Addgene Plasmid# 36206) (Goedhart et al., 2012), and exchanging Rac1 for RhoA (sequence from pcDNA3-EGFP-RhoAQ63L; kind gift of Gary Bokoch, The Scripps Research Institute). In the resulting construct mTurquoise2-NES-2xFKPB'-RhoA Q63L ΔCAAX, 2xFKPB' was replaced by eDHFR using XhoI and XbaI restriction sites and PCR amplification of eDHFR from eDHFR-mCitrine (Chen et al., 2017) using the primers 5′-ctggactcgtacaagatatctcgagctATGATCAGTCT GATTGCGGCGTTAGC-3′ and 5′-tccggatggcagccatggatctagaGGTGGATCCCCGCCGCTC-3′, followed by Gibson assembly. mTurquoise2-NES-eDHFR-RhoA F30L ΔCAAX, mTurquoise2-NES-eDHFR-RhoA wt ΔCAAX and mTurquoise2-NES-eDHFR-RhoA T19N ΔCAAX were generated from this construct by site-directed mutagenesis via the QuikChange approach using primers 5′-CAT AGTCTTCAGCAAGGACCAGCTGCCAGAGGTGTATGTGCC-3′, 5′-CTGTGGGCACATACACCTCTGGCAGCTGGTCCTTGCTGAAG AC-3′ and 5′-CTTTGTGGGACACAGCTGGGCAGGAAGATTATGATCGCCTGAG-3′, 5′-GGGCCTCAGGCGATCATAATCTTCCT GCCCAGCTGTGTCCCAC-3′, and 5′-GCCTGTGGAAAGAACTGCTTGCTCATAGTCTTC-3′, 5′-GAAGACTATGAGCAAGCAGTTC TTTCCACAGGC3′ respectively. TagBFP-Halotag-CAAX and VSVG HaloTag-PARC [mCitrine] were described previously (Chen et al., 2017). mCherry-Zdk1-GEF-H1 C53R was generated by PCR amplification of the pTriEx-mCherry-Zdk1 (Wang et al., 2016) backbone (Addgene Plasmid 81057) using primers 5′-AAAGCTTGCGGCCGCACA-3′ and 5′-gaattcTTTGTCGTCGTCGTCCTTG TAGTC-3′ and PCR amplification of GEF-H1 C53R from EGFP-GEF-H1 C53R (Krendel et al., 2002), with primers 5′-acaaggacgacgacgacaaagaattcATGTCTCGGATCGAATCC-3′ and 5′-gctgtgcggccgcaagctttTTAGCTCTCGGAGGCTAC-3′ followed by Gibson assembly. For pTriEx-NTOM20-CCL-moxBFP-CCL-LOV2 the mVenus fluorophore from pTriEx-NTOM20-mVenus-LOV2 (kind gift of Klaus Hahn, UNC-Chapel Hill School of Medicine, Wang et al., 2016) was replaced by CCL-moxBFP-CCL (CCL: coiled-coil) by PCR amplification of the backbone with primers 5′-ttctgagcagcggttccggatccggtTCCTTGGCTACTACACTTG-3′ and 5′-TCTAGAT TTAAAGTTCGGATCG-3′ and moxBFP (Costantini et al., 2015) (Addgene Plasmid 68064) including CCL-domains with priners 5′-atccgaactttaaatctagaggatctggtagtggttccGCTAGCCTCGCAGCTGCG-3′ and 5′-TCCGGAACCGCTGCTCAG-3′, followed by Gibson assembly. mTurquoise2-eDHFR-GEF-H1 (C53R) was generated by PCR amplification of the mTurquoise2-NES-eDHFR backbone from mTurquoise2-NES-eDHFR-RhoA Q63L ΔCAAX using primers 5′-CTCAATTGTTGTTGTTAACTTG-3′ and 5′-GGATCTA GAGGTGGATCCCCG-3′ and PCR amplification of GEF-H1 C53R including a N-terminal linker region from mCherry-Zdk1-GEF-H1 C53R using primers 5′-ggcggggatccacctctagatccGGTGGTTCTGGTGGTAGC-3′ and 5′-agttaacaacaacaattgagTTAGCTCTCG GAGGCTAC-3′ followed by Gibson assembly. mTurqouise2-eDHFR-LARG was generated by replacing GEF-H1 C53R from mTurquoise2-eDHFR-GEF-H1 C53R with LARG, using NotI and MfeI restriction sites and PCR amplification of LARG from mCherry-LARG (kind gift of Oliver Rocks, MDC Berlin) with primers 5′-aaaatctgtatttccagggcggccgctctggaagtggaAGTGGCACA CAGTCTACTATC-3′ and 5′-ataaacaagttaacaacaacTCAACTTTTATCTGAGTGCTTG-3′, followed by Gibson assembly.

EGFP-NMHCIIA (Wei and Adelstein, 2000) (Addgene Plasmid 11347), mCherry-NMHCIIA (Dulyaninova et al., 2007) (Addgene Plasmid 35687), pTriEx-mCherry-ZdkI and pTriEx-NTOM20-LOV2 (Wang et al., 2016) (Addgene Plasmids #81057 and #81009) plasmids were obtained from Addgene. XL10-Gold® Ultracompetent Cells (Agilent Technologies) were used for transformation of all newly generated plasmid constructs.

Microscopy and chemo-optogenetics

TIRF microscopy was performed primarily on an Olympus IX-81 microscope, equipped with a TIRF-MITICO motorized TIRF illumination combiner, an Apo TIRF 60 × 1.45 NA oil immersion objective and a ZDC autofocus device. On this setup, a quadruple bandpass dichroic mirror (U-M3TIR405/445/514/561, Olympus, Hamburg) was combined with Semrock Brightline emission filters (HC 520/35 and HC 629/53, AHF Analysentechnik, Tübingen), a 405 nm CellR diode laser (100 mW), a 445 nm CellR diode laser (200 mW), a 514 nm OBIS diode laser (150 mW) (Coherent, Inc., Santa Clara, USA) or the 514 nm line of a 400 mW Argon ion laser (model # 543-A-A03, Melles Griot, Bensheim, Germany), and a 561 nm CellR diode laser (100 mW). Photouncaging was performed using the 405 nm laser, which was focused to a spot via the FRAP mode of the TIR-MITICO motorized TIRF illumination combiner. Photouncaging was performed for 400 ms at 180 nW; laser power was measured at the 60x objective. As the perturbation is irreversible, only one pulse was applied to each cell. For detection, an EMCCD camera was used at medium gain without binning. In some experiments, TIRF microscopy was performed on an Eclipse Ti-E (Nikon) inverted microscope with a motorized TIRF Illuminator Unit, an AOTF Laser Combiner and an iXon3 897 single photon detection EMCCD camera. Laser lines used for excitation of EGFP and mCherry were 488 nm and 561 nm, respectively. Images were acquired using an Apo TIRF 100 × 1.49 NA oil immersion objective (Nikon) with an EM-Gain of 50-100 and 2x2 binning. A dual bandpass dichroic mirror (zt488/561rpc) was used in combination with a CSU Quad Dichroic mirror/emission filter set (405/488/568/647 nm). Acquisition was controlled by Andor IQ Software. Both microscopes are equipped with temperature-controlled incubation chambers. Time-lapse live-cell microscopy experiments were carried out at 37°C in CO_2-independent medium (HBSS buffer, 10% FBS, 2 mM L-Glutamine, 10 mM HEPES, 1 mM $MgCl_2$, 1 mM $CaCl_2$) with indicated frame rates.

Simplified molecular activity painting

Molecular activity painting was described in detail previously (Chen et al., 2017). Here, we describe a simplified variant that achieves similar results. In particular, the complexity of the surface immobilization procedure was simplified significantly (see also Figure S2). Here, we coat glass-bottom dishes (8-well LabTek I, VWR) with 0.1% poly-L-lysine hydrobromide (Sigma-Aldrich) at 4°C over night, followed by washing 3 times with DPBS. Adsorbed poly-L-lysine was biotinylated using 1 mg/ml EZ-Link Sulfo-NHS-Biotin (Fisher Scientific) (dissolved in DPBS) for 60 min at room temperature. Subsequently, two solutions were prepared on ice: 1) a streptavidin solution containing 2 μL streptavidin (Serva) (1 mg/ml), 1 μl streptavidin Alexa Fluor 750 (0.1 mg/ml) (Life Technologies) and 12 μl DPBS, and 2) an antibody solution containing 3 μl biotin-labeled anti-VSVG-antibody (1 mg/ml, ab34774, Abcam) and 12 μl DPBS. After washing the dish 3 times with DPBS, all the remaining DPBS was aspirated from the glass surface of the dish. The two solutions were quickly mixed and individual drops of this solution (~2 μl) were added onto the dry dish glass surface to create small circular patterns with a diameter of 1-2mm. After incubation for 2 min the dishes were washed 3 times with DPBS and stored at 4°C with DPBS until cells were added on the same day. This simplified procedure avoids the use of time-consuming specialized covalent DNA surface modification techniques and specialized reagents for DNA-directed immobilization, such as DNA-modified Streptavidin. The fluorescence signal of streptavidin Alexa Fluor 750 facilitated identification of antibody functionalized areas.

Transfected U2OS cells were detached by 20-30 min incubation with 10 mM EDTA (in DPBS, pH 7.4) to avoid digestion of extracellular artificial receptor domains by trypsin. The cell suspension was washed with serum-free media by centrifugation, followed by a second centrifugation step and resuspension in serum-free media and plating onto the antibody-functionalized glass bottom-dishes. After 30 min at 37°C and 5% CO_2 the same volume of 20% FBS (Pan Biotech) containing media was added carefully and cells were allowed to attach for additional 6 h. Covalent labeling for chemo-optogenetic perturbation was performed by incubation with 10 μM of the Nvoc-TMP-Cl photocaged chemical dimerizer (Chen et al., 2017) in HEPES-stabilized imaging medium for 60 min. Excess photodimerizer was removed by three consecutive wash steps with HEPES-stabilized imaging medium and a fourth wash step after an additional 30 min incubation time.

Optogenetic tuning of cytosolic GEF-H1

To control the cytosolic concentration of GEF-H1 in living cells, we used the LOVTRAP system, which was described previously (Wang et al., 2016). In the original method, single pulses at 445nm of varying intensity and duration were used to dissociate the interaction between a mitochondria-targeted LOV domain and a cytosolic protein of interest fused to the dark-state binding domain Zdk1. Here, we used a 100x neutral density filter to reduce the intensity of 422-432 nm light from the MT20 halogen lamp illumination system (Olympus) to enable gradual release of GEF-H1 (C53R) fused to Zdk1 into the cytosol with continuous illumination. To control the level of GEF-H1 release, we increased the output of the halogen lamp between 0% and 100% (5.33 μW) in 8 steps of 400 s via the automated filter wheel built into the MT20 device. GEF-H1 release into the cytosol was monitored by measuring the minimum intensity of the TIRF signal. Intensity measurements via TIRF were variable in subsequent experimental repetitions. Therefore, for analysis that included multiple cells (Figures 3F and S4E), the minimum intensity of the epifluorescence signal was measured in a cell region that was devoid of dense mitochondria structures or the nucleus. Please note that EGFR-GEF-H1 C53R levels shown in Figures 2K and S3E were obtained by a distinct experimental setup and therefore cannot be compared as absolute values with cytosolic GEF-H1 signals shown in panels Figures 3C–3F.

The original pTriEx-NTOM20-LOV2 construct had a tendency to generate aggregates, which made it difficult to achieve efficient recovery of Zdk1-GEF-H1 back to mitochondria. We therefore used an optimized LOVTRAP construct (pTriEx-NTOM20-CCL-moxBFP-CCL-LOV2) for the reversible release of GEF-H1 into the cytosol. Using this optimized construct, recovery of Zdk1-GEF-H1 back to mitochondria was observed in ~50% of all cells (16 of 31 cells from three independent experimental repetitions). Cells that did not show a recovery were excluded from the analysis shown in Figure S4E. For release of Zdk1-GEF-H1, cells where illuminated for 30 min with 57% (2.95 μW) output of the halogen lamp, followed by 10 min with 3 steps of decreasing intensities and 33 min with 0% intensity for recovery (in Figure S4 intensities were normalized to 100%).

Theoretical methods

Minimal ODE model and parameter estimation

The activation and inactivation of Rho is well-known to be mediated by enzymatic reactions that are catalyzed by guanine nucleotide exchange factors (GEFs) and GTPase activating proteins (GAPs) (Hodge and Ridley, 2016), which we implemented via Michaelis-Menten kinetics. The inactive Rho-GDP form resides primarily in the cytosol and active Rho-GTP is primarily localized at the plasma membrane (Garcia-Mata et al., 2011). To keep the number of components minimal, we do not explicitly implement additional mechanisms that are involved in Rho GTPase regulation, such as RhoGDI-mediated shuttling between the active and inactive populations (Garcia-Mata et al., 2011). Activation and inactivation of Myosin (non-muscle Myosin-IIa, MYH9) is mediated by a multi-step enzymatic cascade that involves several kinases, phosphatases, and actin polymerization (Riento and Ridley, 2003). To simplify this complex cascade, we approximated it by a single activating and a single inactivating Michaelis-Menten process. The motor domain of Myosin can bind to the DH domain of GEF-H1 and thereby inactivate the nucleotide exchange activity of this GEF (Lee et al., 2010). This closes a negative feedback loop that can inhibit Rho activation by GEF-H1. As this process is based on a simple interaction, it was implemented by non-enzymatic mass action kinetics. An additional, negative feedback loop that could be mediated by Myo9b (MYO9B) (Graessl et al., 2017) is not likely to play a major role in our model system (U2OS cells), as the endogenous expres-

sion levels of Myo9b (~500 molecules/cell) and its close relative Myo9a (< 500 molecules/cell) are very low, while Myosin-IIa is expressed at a very high level ($1.24*10^6$ molecules/cell) (Beck et al., 2011). Finally, the activation of GEF-H1 by active Rho via plasma membrane recruitment, which closes a positive feedback loop, is based on the interaction between active Rho and the GEF-H1 PH domain (Figure 1). Therefore, this causal relationship is implemented by non-enzymatic mass action kinetics.

On the basis of the biochemical reaction scheme, which is summarized in Figure 2A, we formulated a system of three ordinary differential equations for the variables R(t), G(t), and M(t), that correspond to the active, plasma membrane associated form of RhoA, GEF-H1, and Myosin, respectively. Time is represented by t. In the absence of stochastic noise or spatial inhomogeneity, this system can be postulated as:

$$\frac{dR}{dt} = \frac{k_1 G \cdot (R_T - R)}{K_{m1} + (R_T - R)} - \frac{k_2 \cdot R}{K_{m2} + R} \quad \text{(Equation 1)}$$

$$\frac{dG}{dt} = k_3 (G_T - G) \cdot R - k_4 G \cdot M \quad \text{(Equation 2)}$$

$$\frac{dM}{dt} = \frac{k_5 R \cdot (M_T - M)}{K_{m5} + (M_T - M)} - \frac{k_6 \cdot M}{K_{m6} + M} \quad \text{(Equation 3)}$$

G_T, R_T and M_T are the total concentrations of GEF-H1, Myosin and RhoA, k_1 to k_6 are rate constants and K_{m1}, $K_{m2,}$ K_{m5} and $K_{m6,}$ are Michaelis constants. Total concentrations of GEF-H1 (G_T), RhoA (R_T) and Myosin (M_T) correspond to the sum of the active, plasma membrane-associated and inactive, cytosolic forms and are assumed to be constant over time. Values for these total concentrations in U2OS cells were measured previously (Beck et al., 2011). We varied the total concentration of GEF-H1 (G_T), which was used as a key bifurcation parameter, both analytically and experimentally. The range of GEF-H1 concentrations spanned low levels that are comparable to measured endogenous expression levels of GEF-H1 and related Lbc-types GEFs in U2OS cells (Beck et al., 2011), up to high levels that correspond to increased exogenous expression driven by a strong promoter.

The remaining model parameter values were obtained by a Bayesian fitting approach (Campillo-Funollet et al., 2019; Turkmann et al., 2019). This allows us to estimate and infer the probability distribution for the model parameters on the basis of both prior knowledge and experimental observations. A sample from this probability distribution is a set of parameters for the ODE system. In this work, it is a distribution in an eleven-dimensional space. To evaluate it, we need to solve the ODE system and compute the probability of representing the experimental data with this distribution of parameters. Therefore, it is difficult to perform this directly—as one would do for instance with a one-dimensional Gaussian, by simply computing the density function. An alternative is to use Markov Chain Monte Carlo (MCMC) approaches, which can be used to generate samples from a distribution. This approach takes a large number of samples and generates a histogram, instead of representing the density function. MCMC constructs a chain of parameter sets that overall follow the distribution of interest. The classical MCMC works by generating candidate parameter sets from the prior, and accepting or rejecting them by comparing their relative probabilities. The validation of the samples is done in two steps: first, each chain is checked for convergence, by taking subsamples of the chain and checking that the mean is the same; second, we produce several chains, and compare them with each other in a similar fashion.

Here, we estimated the probability distribution for the unknown model parameters given the experimental measurements shown in Table S1. As prior knowledge, we assumed reasonable ranges for all unknown parameters and represent our initial uncertainty about these parameters as uniform distributions. For any given parameter set that we randomly sampled from these distributions, we first analyzed the stability of the ODE system steady state and discarded parameter sets that did not generate oscillations when increasing G_T. Therefore, we used a constrained uniform prior. We approximated the posterior distribution for the parameters using a parallelized MCMC that allows solving many instances of the ODE system in parallel in a High-Performance Computing (HPC) environment (Campillo-Funollet et al., 2019). We verified the convergence of the algorithms by running 15 independent chains with random initial values. All chains converged in mean to the same values, and exhibit comparable second moments. Therefore, we conclude that the optimal set of parameter values is unique. To perform the posterior analysis, we removed the initial 10% of samples—about 10^5 samples—and thinned the rest of the chain to obtain 10^4 independent values. We computed the most probable values, i.e., the modes of the posterior distributions, and credible regions for the parameters by finding regions that contain 95% of the probability mass (Table S3). Posteriori analysis also showed that all chains converged to the same modes. As we add an additional constraint to the prior distributions (see above), the highest probability for many parameters is not centered in the interval, but instead near a boundary of the parameter range that can generate oscillations. The initial parameter ranges that were used for fitting are given in Table S2.

Bifurcation analysis

The ODE system (Equations 1, 2, and 3) that was parameterized by the MCMC approach, was investigated by bifurcation analyses using the software package XPPauto (*XPPauto webpage*, http://www.math.pitt.edu/~bard/xpp/xpp.html). The bifurcation diagram for Rho shown in Figure 2I provided the estimations for maximal and minimal concentrations of active Rho in the oscillatory regime (0.00 to 0.34×10^6 molecules/cell), which are shown in Table 1. The maximal concentration of inactive Rho was obtained by

subtracting the minimal concentration of active Rho from the total Rho concentration (R_T). The values for the minimal and maximal concentrations of active Myosin (0.03 to 0.24 × 10^6 molecules/cell) and the maximal concentration of inactive Myosin were obtained from an analogous bifurcation diagram for the Myosin component and from the total Myosin concentration (M_T).

Simulation of the SDE system

To investigate the influence of partially stochastic biochemical and/or mechanical inputs into the Myosin component, we extended Equation 3 of the ODE system described above by an additive noise term σ dW(dt). The resulting system of stochastic differential equations (SDE) is defined by the following equations:

$$dR = \left(\frac{k_1 G \cdot (R_T - R)}{K_{m1} + (R_T - R)} - \frac{k_2 \cdot R}{K_{m2} + R}\right) dt \qquad \text{(Equation 4)}$$

$$dG = (k_3 (G_T - G) \cdot R - k_4 G \cdot M) dt \qquad \text{(Equation 5)}$$

$$dM = \left(\frac{k_5 R \cdot (M_T - M)}{K_{m5} + (M_T - M)} - \frac{k_6 \cdot M}{K_{m6} + M}\right) dt + \sigma \cdot dW$$
$$with: \ dW = \mathrm{r} \cdot \sqrt{dt}. \qquad \text{(Equation 6)}$$

Here, r are normally distributed random numbers with mean value 0 and standard deviation of 1. σ corresponds to the term $Noise_{Myosin}$ and was set between 0 (no noise) and 0.0025. All other parameters were kept identical to the ODE system Equations 1, 2, and 3. Simulations were performed by the Euler-Maruyama method (Platen, 1999), which calculates the change in the active Rho, GEF and Myosin components with discrete, constant time steps. In our simulations, we used a time step of 0.05 s. To prevent noise-driven crossing of variables below the value 0, an adaptive time step algorithm was used that reduced the time step by 50% until positive numbers were obtained.

Cellular automata simulations

The spatio-temporal simulations were performed using cellular automata as described previously (Schmick et al., 2014). In this model we consider a 2-dimensional surface that represents local subcellular regions of the plasma membrane and the cytosol. This surface is subdivided into discrete spatial regions that are referred to as "cells." We consider reactions between the system components inside each individual cell, and their diffusion by simulating mass transfer between cells. Simulations were initialized with a homogeneous distribution of reaction components, with a low-level of noise that was added to each cell. Simulations were performed in a 100 × 100 cell array corresponding to 50μm x 50μm, with a time step of 0.25 s and cyclic boundary conditions. Reactions were implemented using the same equations used for the SDE model above, with the modification that inactive and active species were implemented separately with corresponding terms for their diffusion, resulting in the following equations:

$$dR_{active} = \left(\frac{k_1 G_{active} \cdot R_{inactive}}{K_{m1} + R_{inactive}} - \frac{k_2 \cdot R_{active}}{K_{m2} + R_{active}} + D_{R,active} \cdot \nabla^2 R_{active}\right) dt \qquad \text{(Equation 7)}$$

$$dG_{active} = (k_3 G_{inactive} \cdot R_{active} - k_4 G_{active} \cdot M_{active} + D_{G,active} \cdot \nabla^2 G_{active}) dt \qquad \text{(Equation 8)}$$

$$dM_{active} = \left(\frac{k_5 R_{active} \cdot M_{inactive}}{K_{m5} + M_{inactive}} - \frac{k_6 \cdot M_{active}}{K_{m6} + M_{active}} + D_{M,active} \cdot \nabla^2 M_{active}\right) dt + \sigma \cdot dW_t \qquad \text{(Equation 9)}$$

$$dR_{inactive} = -\left(\frac{k_1 G_{active} \cdot R_{inactive}}{K_{m1} + R_{inactive}} - \frac{k_2 \cdot R_{active}}{K_{m2} + R_{active}} + D_{R,inactive} \cdot \nabla^2 R_{inactive}\right) dt \qquad \text{(Equation 10)}$$

$$dG_{inactive} = -(k_3 G_{inactive} \cdot R_{active} - k_4 G_{active} \cdot M_{active} + D_{G,inactive} \cdot \nabla^2 G_{inactive}) dt \qquad \text{(Equation 11)}$$

$$dM_{inactive} = -\left(\frac{k_5 R_{active} \cdot M_{inactive}}{K_{m5} + M_{inactive}} - \frac{k_6 \cdot M_{active}}{K_{m6} + M_{active}} + D_{M,inactive} \cdot \nabla^2 M_{inactive}\right) dt - \sigma \cdot dW_t$$
$$with: dW_t = \mathrm{r} \cdot \sqrt{dt}. \qquad \text{(Equation 12)}$$

Here, $D_{R,active}$, $D_{R,inactive}$, $D_{G,active}$, $D_{G,inactive}$, $D_{M,active}$ and $D_{M,inactive}$ are the diffusion coefficients for the active, plasma membrane-associated and the inactive cytosolic states of the Rho GTPase, GEF-H1 and Myosin components. For simplification, we assumed that the cytosolic species are always inactive and plasma membrane-associated species are always active. The diffusion coefficients of the active plasma membrane-associated and inactive cytosolic Rho species were based on previous measurements of Rho mobility (Weitzman, 2013). Here, the predominant, fast diffusing fractions were used (inactive Rho D = 9.28 $\mu m^2/s$; active Rho D = 0.28 $\mu m^2/s$). Experimental data were also available for the diffusion of a cytosolic non-muscle Myosin-II isoform (NMY-2; D = 0.9 $\mu m^2/s$) (Petrásek et al., 2008). In that study, plasma membrane-associated Myosin-II did not show typical diffusive behavior, however, its measured mobility was much lower compared to the cytosolic Myosin-II isoform (Petrásek et al., 2008). In our experiments, Myosin dependent contractile flow points toward the highest Myosin concentrations and thus operates opposite to diffusion. We therefore used a very slow diffusion coefficient for the plasma membrane-associated Myosin component (D = 0.03 $\mu m^2/s$). Diffusion of GEF-H1 in the cytosol was measured using fluorescence correlation spectroscopy (FCS) as described earlier and found to be indistinguishable from diffusion of cytosolic Rho. Diffusion of plasma membrane-associated GEF-H1 was assumed to be similar to diffusion of plasma membrane associated RhoA due to their interaction. In these simulations, the non-negativity of solutions is enforced.

QUANTIFICATION AND STATISTICAL ANALYSIS

Analysis of signal network dynamics in cells

Kinetics of Rho, GEF or Myosin fluorescence signal changes after perturbations via chemo-optogenetics were measured at the local perturbation region as described previously (Chen et al., 2017). The fluorescence signal profiles of the measured perturbation and response signals shown in Figures 2D and S3D are normalized to minimum and maximum values, and therefore absolute amounts of signals cannot be compared.

As unbiased measures for local signal network dynamics in all central regions of the cell attachment area, we determined the average peak amplitude and the standard deviation of local Rho signal using ImageJ as described previously (Graessl et al., 2017). The coefficient of variation of interpeak distance of Rho activity pulses in U2OS cells was determined using MATLAB and ImageJ via a similar, unbiased approach. In brief, image sequences were obtained at a frame rate of 3 per minute and scaled down by a factor of 15 using the averaging command in ImageJ to reduce noise. Individual cells in image sequences were isolated, masked, and corrected for background intensity. The peripheral pixels were removed using a single application of the binary erode filter with neighborhood count of 1 to avoid measurements of signal changes that originate from dynamic cell protrusion and to perform analysis of signal dynamics in central cell attachment areas. In each pixel, intensity changes were determined over time and analyzed as follows: The baseline was corrected by fitting and subtracting a 6-th order polynomial. Peaks were identified in baseline corrected time series using the MATLAB findpeaks function with a prominence threshold of 40% of the maximum and minimum values of the timeseries. Only time series that contain at least 10 peaks were included in the subsequent analysis. To obtain the coefficient of variation, the time interval between subsequent, detected peaks was determined and the standard deviation of all intervals was divided by its mean value. The oscillation frequency was also measured in each pixel of the masked and scaled down image sequences by applying the fast Fourier transform function fft that is built into MATLAB. The corresponding GEF-H1 expression levels were measured by calculating the average of the widefield signal in the entire cell at the start and the end of the video.

Analysis of dynamics in simulations

The baseline of simulations converged to a stable average value, and therefore correction via polynomial fitting was not necessary. Instead, peaks were directly identified in the raw time series using the MATLAB findpeaks function with a prominence threshold of 10% of the maximum and minimum values of the timeseries. Detected peaks were used to determine the coefficient of variation of interpeak distance, as well as the average and maximal peak amplitude. The spatial width of Rho activity pulses in cellular automata simulations were measured using the ICSMATLAB image correlation spectroscopy functions (https://github.com/stevekochscience/Image-Correlation-Spectroscopy). In brief, the 2D image correlation functions were calculated using corrfunction for each frame of a 100x100 pixel time series, and the mean correlation function was determined by averaging each pixel of the resulting correlation functions. The center pixels were selected via the autocrop function and fitted to a 2D Gaussian using the gaussfit function. The average of the two fitted sigma values was calculated and the full width at half maximum was determined by multiplying the average sigma with $2 \times \text{sqrt}(2 \times \ln 2)$.

Statistical Analysis

All statistical analyses were computed using Prism (GraphPad). Plots were generated with Prism or XPP (*XPPauto webpage*, http://www.math.pitt.edu/~bard/xpp/xpp.html). Image panels were prepared with ImageJ (Rasband, W.S., ImageJ, U. S. National Institutes of Health, Bethesda, Maryland, USA, https://imagej.nih.gov/ij/, 1997–2018.). The type of statistical tests, number and type of repeats, precision measures, and significance levels are indicated in the respective figure legends.

Cell Reports, Volume *33*

Supplemental Information

Optogenetic Tuning Reveals Rho Amplification-Dependent Dynamics of a Cell Contraction Signal Network

Dominic Kamps, Johannes Koch, Victor O. Juma, Eduard Campillo-Funollet, Melanie Graessl, Soumya Banerjee, Tomáš Mazel, Xi Chen, Yao-Wen Wu, Stephanie Portet, Anotida Madzvamuse, Perihan Nalbant, and Leif Dehmelt

Supplemental Information

Supplemental Figures:

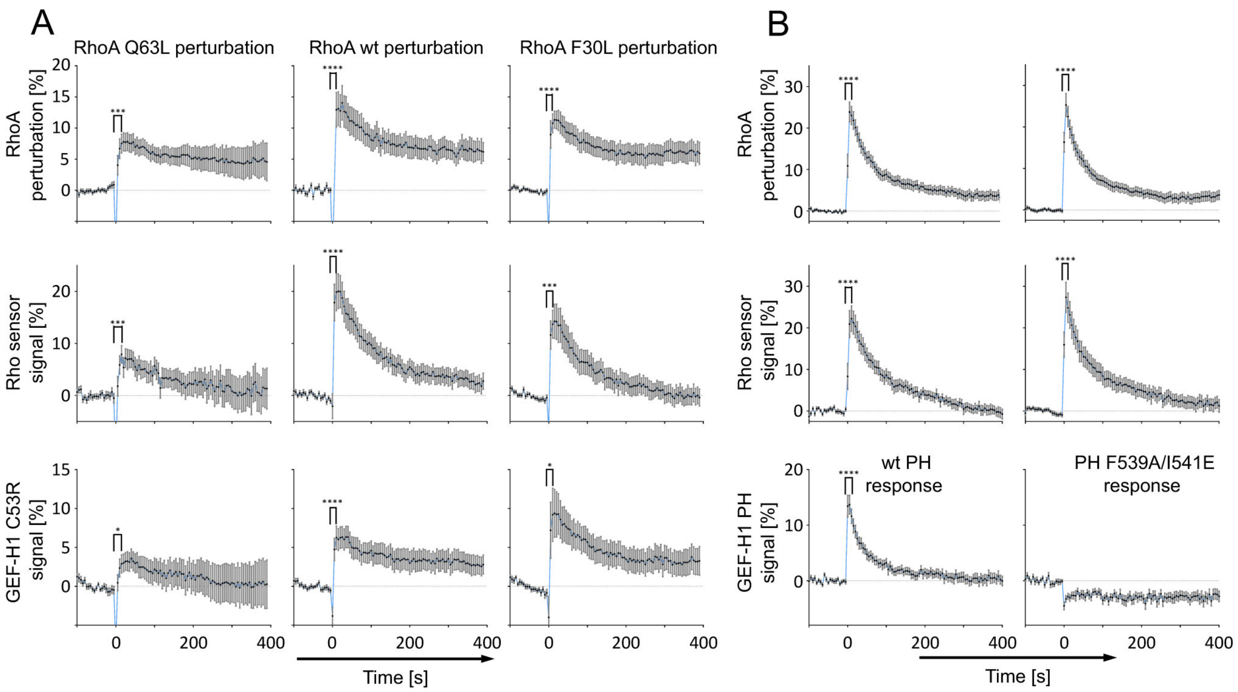

Supplemental Figure 1 (related to Figure 1): Direct investigation of Rho-dependent GEF-H1 plasma membrane recruitment. A: Quantification of the RhoA plasma membrane recruitment and Rho activity sensor/GEF-H1 co-recruitment. **B:** Quantification of GEF-H1 PH domain co-recruitment with active RhoA Q63L. F539A/I541E: PH domain mutant that is deficient in its interaction with active Rho. (**A-B**: % increase above average intensity before photoactivation at t=0s with standard error of the mean (SEM); $n \geq 14$ (**A**) or $n \geq 21$ (**B**) cells from three experiments; ****, $P \leq 0.0001$; ***, $P \leq 0.001$; **, $P \leq 0.01$; *, $P \leq 0.05$; paired t test before and 10 s after photoactivation);

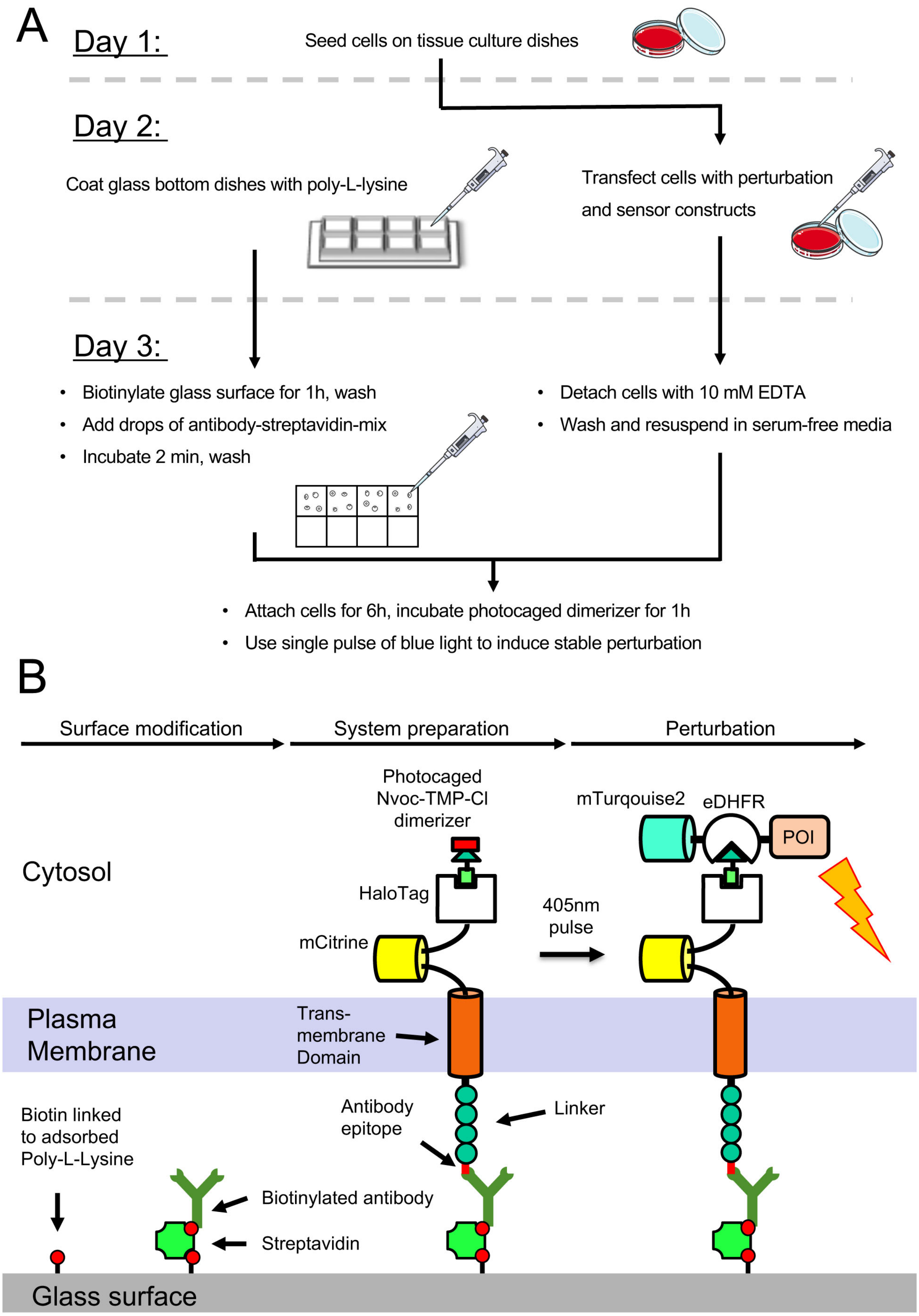

Supplemental Figure 2 (related to Figure 2): Schematic summary of improved "Molecular Activity Painting" technique to acutely introduce stable perturbations at the plasma membrane of living cells. A: Experimental workflow and time considerations. **B**: Schematics for step-wise construction of perturbation system. This figure was created using Servier Medical Art templates, which are licensed under a Creative Commons Attribution 3.0 Unported License; https://smart.servier.com

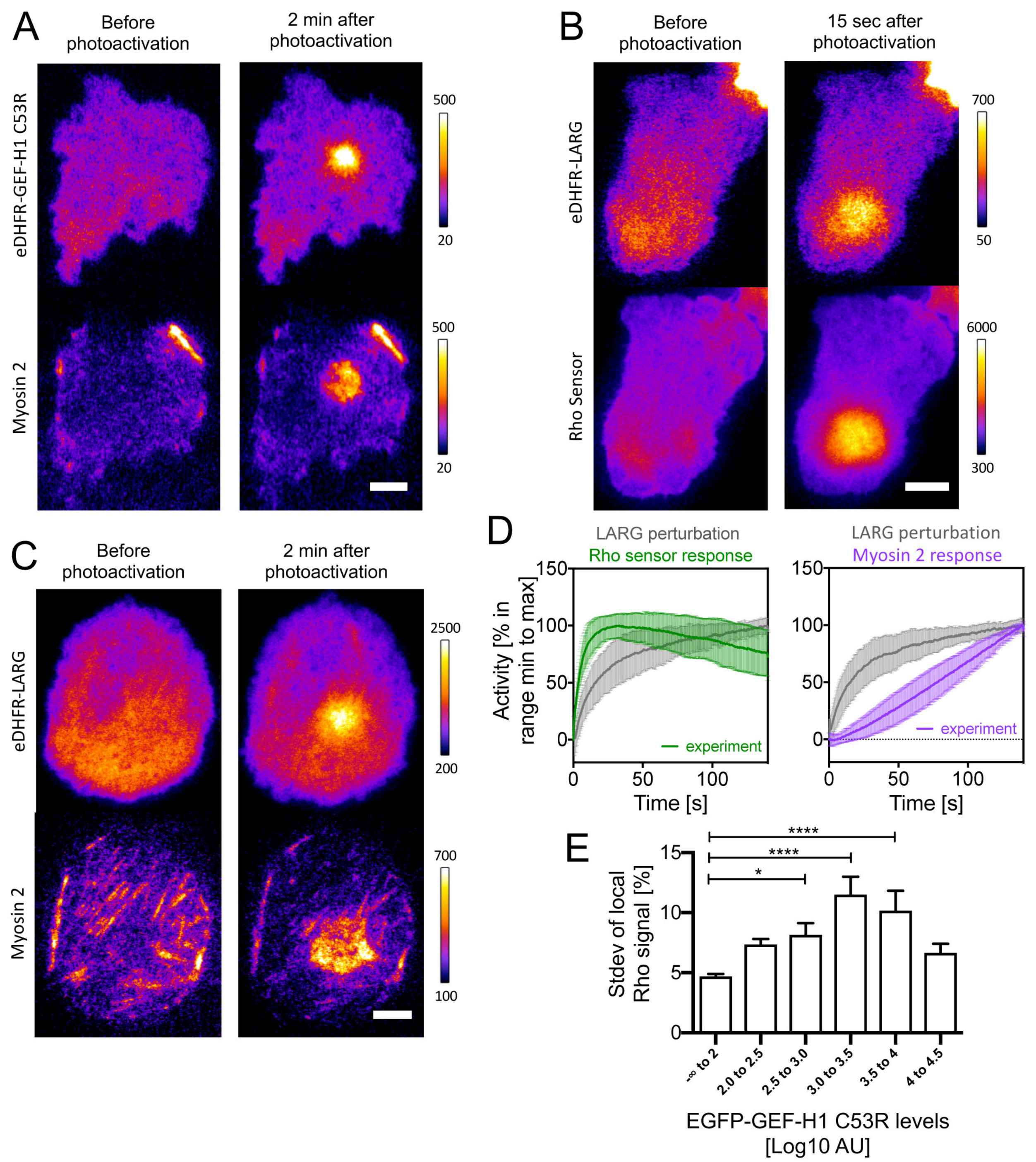

Supplemental Figure 3 (related to Figure 2): Rho activity and myosin cell cortex recruitment response to Lbc-type GEF perturbation. A: TIRF images of immobilized GEF-H1 C53R perturbation and Myosin response (see also Video 2). **B-C:** TIRF images of immobilized LARG perturbation and Rho activity sensor **(B)** or Myosin **(C)** response (see also Video 2). **D:** Kinetics of Rho activity sensor and Myosin cell cortex recruitment response to acute chemo-optogenetic LARG perturbations ($n \geq 23$ cells from three experiments, mean with SEM). **E**: Dependence of Rho activity dynamics on the effective cytosolic concentration of GEF-H1 C53R. Activity dynamics were measured by determining the standard deviation of the local Rho activity sensor signal (n=124 cells from three experiments; mean and SEM; One way ANOVA, Dunnett's post test, *: $P<0.05$, ****: $P<0.0001$). % indicates percentage of mean intensity. Scale bars: 10µm.

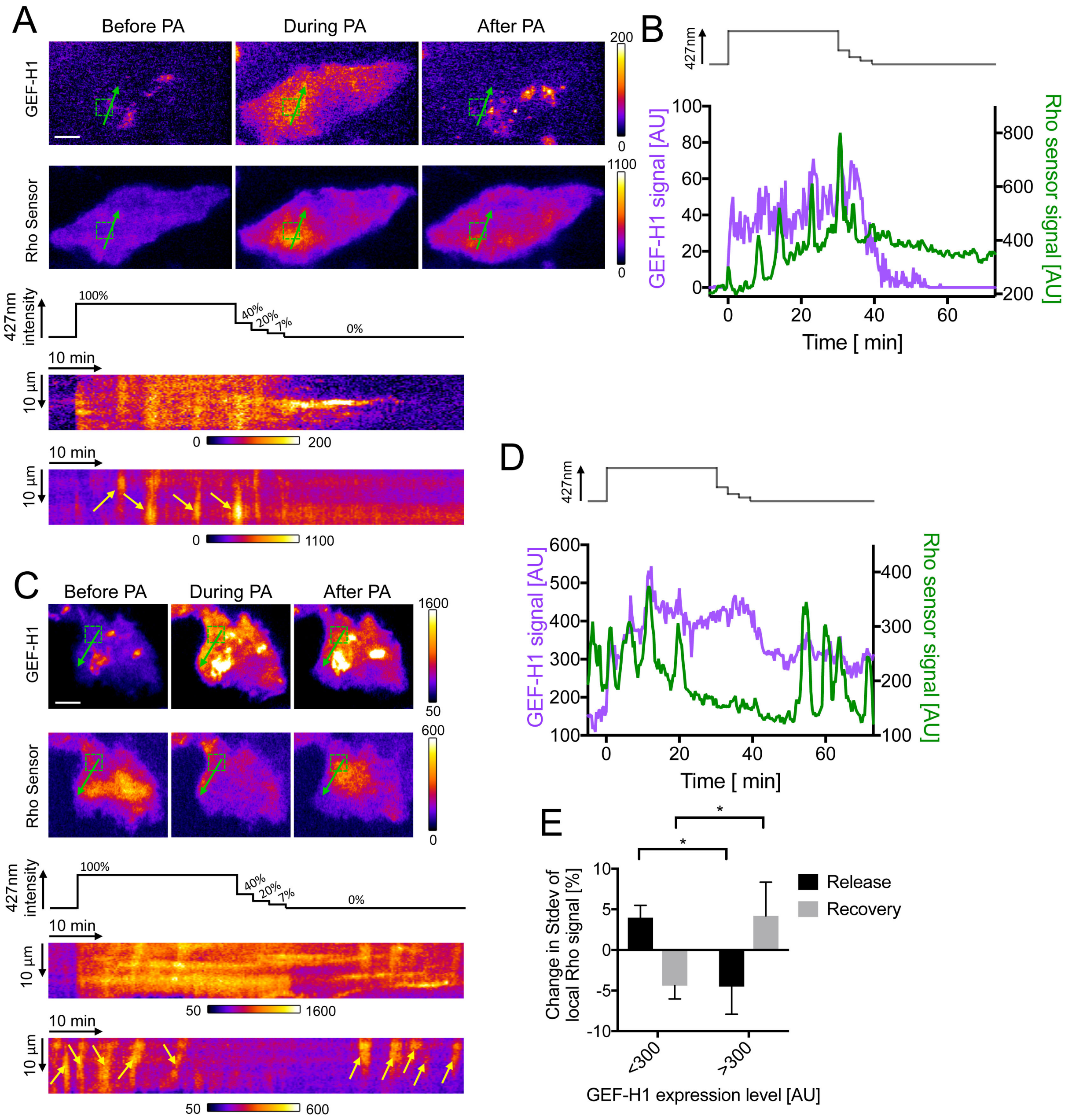

Supplemental Figure 4 (related to Figure 3): GEF-H1 concentration-dependent switching of Rho activity dynamics by reversible optogenetic tuning. A-D: Analysis of two representative cells that demonstrate either reversible activation **(A,B)** or reversible saturation **(C,D)** of Rho activity dynamics during subsequent increase and decrease of the effective cytosolic concentration of GEF-H1 via photoactivation (PA) of the LOVTRAP system. **A,C:** Top: TIRF images of mCherry-Zdk1-GEF-H1(C53R) and the Rho sensor (see also Video 5). Bottom: Kymographs corresponding to green arrows in top panel. Yellow arrows point to Rho activity pulses or Rho activity waves. **B,D:** Cytosolic mCherry-Zdk1-GEF-H1(C53R) levels obtained as the minimum signal in green boxed regions in **A** or **C**, and measurements of the Rho activity sensor signal over the time course of the experiment. **E:** Local standard deviation of Rho activity signals at low (<300) or high (>300) levels of mCherry-Zdk1-GEF-H1(C53R) from all analyzed cells (n = 16 cells from three experiments, mean and SEM; unpaired t-test, *: P<0.05). Columns represent the change of the average local standard deviation of Rho activity signals induced by the release of GEF-H1 from mitochondria (comparing the time ranges -5 to 0 min before PA with 25 – 30 min during PA) or induced by the recovery of GEF-H1 to mitochondria (comparing the time ranges 25 – 30 min during PA with 25 – 30 min after PA). % indicates percentage of mean intensity. PA: photoactivation. Scale bars: 10µm.

Supplemental tables

Experimental measurement	Measured values
GEF-H1 perturbation rate constant (mono-exponential fit to grey curve in Figure 2D)	0.0789 +/- 0.006 s^{-1} (with Rho sensor; left panel)<br>0.0397 +/- 0009 s^{-1} (with Myosin; right panel)
Rho and Myosin response kinetics	Simulations were fitted to all time points shown in Figure 2D
Period of Rho activity pulses	248 +/- 95 s (n=10 cells; in the presence of active GEF-H1 C53R, this work)
Time shift Myosin after Rho	39.5 +/- 14.7 s (n=37 cells; Graessl et al.)
Time shift Rho after GEF-H1	2.5 +/- 5.6 s (n=68 cells; Graessl et al.)

Supplemental Table 1: Experimental data used to parameterize the ODE system (related to Figure 2). The ODE system is given in Eq.(1-3).

Parameter	Lower bound for fitting	Upper bound for fitting
Concentrations		
in 10^6 molecules/cell:		
G_T (oscillatory regime)	0.142	20
G_T (perturbation/Rho response)	0.0142	1
G_T (perturbation/Myosin response)	0.0142	1
R_T (all regimes; fixed value based on[12])	0.443	0.443
M_T (all regimes; fixed value based on[12])	1.24	1.24
Rate constants:		
k_1/K_{m1} in $s^{-1}(10^6$ molecules/cell$)^{-2}$	0.316	31.6
k_2/K_{m2} in $s^{-1}(10^6$ molecules/cell$)^{-1}$	0.15	150
k_3 in $s^{-1}(10^6$ molecules/cell$)^{-2}$	0.15	15
k_4 in $s^{-1}(10^6$ molecules/cell$)^{-2}$	0.015	15
k_5/K_{m5} in $s^{-1}(10^6$ molecules/cell$)^{-2}$	0.005	5.00
k_6/K_{m6} in $s^{-1}(10^6$ molecules/cell$)^{-1}$	0.000844	2.67
Michaelis constants in 10^6 molecules/cell:		
K_{m1}	0.0475	47.5
K_{m2}	0.01	10
K_{m5}	0.003	3.00
K_{m6}	0.0563	5.63

Supplemental Table 2: Parameters of the ODE system and their prior distributions for fitting to experimental data (related to Figure 2). The ODE system is given by Eq.(1-3). The distributions are defined by the given ranges with equal probability.

Physical parameter	Estimated value (mode of distribution)	95% lower bound	95% upper bound
Total concentrations in 10^6 molecules/cell:			
GEF-H1 (G_T); oscillatory regime	0.179	0.178	4.64
GEF-H1 (G_T); perturbation regime; Rho response	0.0858	0.0491	0.175
GEF-H1 (G_T); perturbation regime; Myosin response	0.233	0.0203	0.234
Rho (R_T); all regimes; fixed value based on[12]	0.443		
Myosin (M_T); all regimes; fixed value based on[12]	1.24		
Rate constants:			
k_1/K_{m1} in $s^{-1}(10^6$ molecules/cell$)^{-1}$	3.88	1.09	5.37
k_2/K_{m2} in s^{-1}	2.04	0.880	3.83
k_3 in $s^{-1}(10^6$ molecules/cell$)^{-1}$	1.19	0.723	3.34
k_4 in $s^{-1}(10^6$ molecules/cell$)^{-1}$	3.98	3.97	14.9
k_5/K_{m5} in $s^{-1}(10^6$ molecules/cell$)^{-1}$	0.417	0.074	0.718
k_6/K_{m6} in s^{-1}	0.00509	0.000904	0.00510
Michaelis constants in 10^6 molecules/cell:			
K_{m1}	2.42	0.0886	2.43
K_{m2}	0.0745	0.0741	0.564
K_{m5}	0.014	0.00733	0.0658
K_{m6}	0.786	0.258	1.83

Supplemental Table 3: Parameters of the ODE system and their estimations obtained via fitting to experimental data (related to Figure 2). The ODE system is given by Eq.(1-3). The values correspond to the mode of the posterior distribution. The lower and upper bounds correspond to the highest posterior density region, containing 95% of the posterior probability mass.
